# A compartmental model for simulating the gut-brain axis in gastric function regulation

**DOI:** 10.3389/fphys.2026.1727491

**Published:** 2026-06-03

**Authors:** Shannon Q. Fernandes, Mayuresh V. Kothare

**Affiliations:** Department of Chemical and Biomolecular Engineering, Lehigh University, Bethlehem, PA, United States

**Keywords:** autonomic nervous system, compartmental modeling framework, computationally inexpensive model, gut-brain axis, vago-vagal loop

## Abstract

**Introduction:**

Gastric function is regulated by the gut-brain axis, which integrates vagal and enteric nervous system (ENS) pathways. The parasympathetic circuit within the vagal pathway promotes digestion by stimulating peristaltic activity and relaxing the pyloric sphincter (PS) through motor and sensory neurons. In contrast, the sympathetic pathway inhibits digestion by suppressing peristalsis and constricting the PS, highlighting the complex neural coordination involved in gastric regulation.

**Methods:**

We introduce a novel mathematical model of the gut-brain axis using a computationally efficient compartmental modeling framework. The model simulates the vagal and ENS pathways and their corresponding effects on gastric function to enhance our understanding of gut-brain axis regulation. We employ the Michaelis-Menten equation with a Hill coefficient to capture neurotransmitter release at neuromuscular junctions by stimulation of motor neurons and its effects on gastric cells. Motor, or efferent, neurons are modeled for three key stomach regions: the fundus, which exhibits tonic activity; the antrum, which exhibits phasic activity; and the PS, which exhibits both tonic and phasic activity. Thus, the stomach is represented as a three-compartment model. The stomach model extends our previous work by incorporating passive stress and dynamic changes in stomach geometry. Sensory, or afferent, inputs are represented through linear equations that account for chemo- and mechanoreceptor activity, while a binary variable captures the sympathetic response. Afferent and efferent firing rates are linked via fitted curves to effectively close the gut-brain axis feedback loop, borrowing from a similar approach used to model cardiovascular regulation.

**Results:**

The simulation results align with physiological observations, demonstrating inhibitory digestive activity during sympathetic responses and excitatory activity, such as gastric emptying, during parasympathetic responses. During gastric emptying, the interstitial cells of Cajal activity shows constant amplitude for low to medium gastric volumes but exhibits an increase in amplitude at very high gastric volumes. Furthermore, gastric emptying rates decrease with high-calorie liquids due to PS regulation.

**Discussion:**

The flexibility of the model allows for future enhancements based on newly discovered signaling pathways in gut-brain circuitry. The computational efficiency of the model suggests its potential use in developing vagal stimulation therapies for gastrointestinal disorders using closed-loop model-based control.

## 1 Introduction

Neural control of the gastrointestinal (GI) system integrates both extrinsic and intrinsic inputs. Gastric function is predominantly regulated by extrinsic inputs from the central nervous system (CNS), with the vagus nerve activity playing a pivotal role in mediating CNS influence on the stomach (Wood, 1994; Travagli et al., 2006; Goyal and Hirano, 1996). Intrinsic control mechanisms, including the submucosal and myenteric plexuses, as well as interstitial cells of Cajal (ICC), also contribute significantly to gastric motility regulation. The coordinated interplay among ICC, smooth muscle cell (SMC), enteric nervous system (ENS), and vagal inputs establishes the patterns necessary for proper gastric function (Travagli and Anselmi, 2016).

The CNS modulates gastric function via the dual influence of parasympathetic and sympathetic pathways, which cooperate to regulate digestive activities. These pathways primarily originate from neural circuits in the caudal brainstem. The sympathetic pathway involves cholinergic preganglionic neurons that originate in the intermediolateral column of the thoracic spinal cord. These neurons project to postganglionic neurons, which innervate the enteric plexus, a localized neural network within the stomach. The sympathetic pathway primarily modulates gastric inhibition by suppressing cholinergic vagal inputs to postganglionic neurons (Travagli et al., 2006; Cervi et al., 2014). This pathway is activated during “fight or flight” responses, highlighting its role in stress-induced gastric modulation (Bhatia and Tandon, 2005).

The parasympathetic pathway employs both excitatory and inhibitory signaling to regulate various gastric functions, including promoting gastric emptying (Browning and Travagli, 2014). Parasympathetic control is mediated through the dorsal motor nucleus of the vagus (DMV), which governs the vagovagal reflex. The parasympathetic neurons in the DMV consist of cholinergic preganglionic neurons projecting to postganglionic neurons within the enteric plexus. These postganglionic neurons, which can be either cholinergic or non-adrenergic, non-cholinergic (NANC), provide excitatory or inhibitory signals to the stomach.

Sensory signaling from the gut to the brainstem involves mechano- and chemo-sensitive inputs transmitted via vagal afferent fibers to the nucleus tractus solitarii (NTS). The NTS processes these inputs and relays them to the DMV through direct or indirect pathways. Indirect pathways integrate signals from limbic and hypothalamic regions, modulating the reflexive output to regulate gastric function (Brookes et al., 2013; Berthoud et al., 2004). Vagal efferent fibers from the DMV project back to the gut, controlling gastric motility. This bidirectional communication between the gut and brainstem, known as the vagovagal reflex, is crucial for maintaining gastric homeostasis (Travagli and Anselmi, 2016).

Notably, the synaptic connections between the NTS and DMV in the brainstem are not static but exhibit plasticity. Previous studies (Browning and Travagli, 2014; Kentish and Page, 2014; Travagli et al., 2006) have shown that this plasticity allows the synaptic circuitry to adapt and fine-tune gastric motor activity in both physiological and pathological states, depending on sensory input. This adaptability underscores the complexity and significance of neural control in gastric function.

The vagovagal reflex is crucial, as disruptions in vagal sensory-motor function can lead to GI disorders. Evidence of this was reported in (Tack et al., 2003; Delgado-Aros et al., 2004), where patients with functional dyspepsia exhibited altered vagal function characterized by reduced gastric compliance and impaired gastric emptying. These findings suggest that impaired vagal regulation may underlie many functional GI disorders (Travagli et al., 2006).

Despite the central role of neural regulation, gastric function is also strongly shaped by region-specific physiological behavior. Experimental and clinical studies demonstrate that the fundus (proximal stomach) primarily exhibits tonic behavior associated with storage and gastric accommodation through distension and regulation of intragastric pressure (IGP) (Azpiroz and Malagelada, 1985; Kindt and Tack, 2006). The antrum generates rhythmic phasic peristaltic contractions responsible for mixing and propulsion of gastric contents (Armitage and Dean, 1963; Oberhelman and H., 1966; O’Connor and O’Moráin, 2014). The pyloric sphincter (PS) functions as a flow-regulating valve, where basal tone and coordinated phasic activity determine gastric outflow and emptying dynamics (Wijnhoven et al., 1998; Richardson et al., 2023; Lu et al., 2022; Goyal et al., 2019). Computational and biomechanical modeling studies further support this functional regionalization and demonstrate that these regions require distinct mechanical and electrophysiological representations (Klemm et al., 2020; Fernandes et al., 2024; Li and Jin, 2021; Ferrua and Singh, 2010).

Nevertheless, existing mathematical models provide only partial representations of vago-vagal loop pathways and their regulatory effects on gastric function. Prior modeling efforts have typically focused on individual components of the gut-brain control hierarchy, including gastric cell electrophysiology (Corrias and Buist, 2007, 2008; Lees-Green et al., 2014), whole-organ gastric mechanics (Fernandes et al., 2024; Du et al., 2010; Klemm et al., 2020; Cheng et al., 2007), enteric nerve physiology (Chambers et al., 2008, 2011, 2014a, 2014b; Miftahof et al., 2009), and enteric neural inputs driving distal antral contractions (Athavale et al., 2024a). Despite increasing recognition of the gut–brain axis and its influence on gastric regulation, there has not been a systematic attempt to build a systems-level model of the entire gut-brain loop that integrates these individual components.

Our aim in this study was to develop a computational model of the gut-brain axis as it pertains to gastric motility and emptying. Using a compartmental modeling framework, we leveraged a phenomenological systems biology approach to ensure a flexible yet computationally efficient implementation. This model integrates seamlessly with our previously established compartmental framework for gastric function (Fernandes et al., 2024) and provides insight into the dynamic interactions between neural and gastric systems. The flexibility of the model allows for future enhancements to the model based on newly discovered signaling pathways, and experimental data emerging from clinical and animal studies of the gut-brain circuitry. Moreover, its computational efficiency ensures that the model can be used as a “digital twin” in advancing closed-loop therapeutic interventions such as vagal nerve stimulation for treating gastric diseases using concepts from model-based control theory (Yao and Kothare, 2023).

## 2 Methods: mathematical modeling

### 2.1 Modeling the autonomic nervous system regions involved in gastric function regulation: an overview

In this study, the vago-vagal signaling involved in gastric regulation is modeled for the human stomach by incorporating pathways derived from animal models that share anatomical and functional similarities with human vagal circuits. Previous studies (Corrias and Buist, 2008; Athavale et al., 2024a; Cao et al., 2022; Pelot et al., 2020; Lu et al., 2020) have demonstrated that species such as pigs and rats exhibit comparable vagal control of gastric electrophysiology, making them valuable for understanding the vago-vagal reflex loop in humans. However, certain interspecies differences exist, particularly in gastric electrophysiology. For instance, the ICC generate “slow waves” at approximately 4 cycles per minute (cpm) in rats, compared to about 3 cpm in both humans and pigs as reported in as reported in both experimental and experimental inspired computational studies papers (Corrias and Buist, 2007; Athavale et al., 2024b; Cao et al., 2022). These baseline frequency differences, along with other species-specific parameter adjustments, have been accounted for in the present model to enable a physiologically consistent translation of animal data to human physiology. The focus is primarily on the parasympathetic pathways, as these are the dominant contributors to gastric motility and are the most extensively characterized in prior experimental studies.

The mathematical model of the vagal brain-gut axis circuitry controlling gastric function is structured into several components, which are discussed in the following subsections. The first subsection outlines the efferent vagal pathways, which mediate gastric motor responses through excitatory and inhibitory signaling. The second subsection develops equations for afferent pathways, capturing sensory input from chemoand mechanoreceptors. The third subsection examines the interneuronal connections within the brain, specifically between the afferent and efferent vagal fibers, for instance, the NTS and DMV interneuron connection. Finally, the fourth subsection integrates the derived pathways to model the parasympathetic and sympathetic circuits governing gastric motility and emptying, offering a unified framework for modeling the gut–brain axis.

The stomach is modeled using a compartmental framework as proposed in our previous study (Fernandes et al., 2024). The framework divides the stomach into three compartments: the fundus, antrum, and PS, represented by the subscripts *w* = 1,2,3, respectively. Unlike the fixed geometry assumed in the previous model, the present study introduces dynamic compartmental geometry, which accounts for changes in gastric volume during motility and emptying. This enhancement provides a more realistic representation of gastric behavior.

Passive stress models, dependent on gastric volume, are integrated into the compartmental framework to capture the biomechanical properties of the stomach. The dynamic volume is constrained between 0.08 L (empty stomach) and 1.2 L (full stomach), consistent with physiological observations reported in prior studies (Pal et al., 2004; Li and Jin, 2021; Du et al., 2010; Karnul and Murthy, 2022).The parameters for the model used in this study are summarized in **Tables 1**, **2**. For ease of readability, the main equations are included in the paper and the mathematical details of the modeling steps are provided in the **Supplementary Materials Section**.

**Table 1:** Neural model parameters for simulating the gut–brain axis in gastric function regulation.

Parameter	Value	Unit	Reference
*A*_[*ACh*]*,tnc,*1_	1031.31	µM	**Supplementary Material Section 1.2**
*A*_[*ACh*]*,tnc,*3_	601.18	µM	**Supplementary Material Section 1.2**
*B*_[*ACh*]*,tnc,*1_	165.13	Hz	**Supplementary Material Section 1.2**
*B*_[*ACh*]*,tnc,*3_	379.05	Hz	**Supplementary Material Section 1.2**
*N*_[*ACh*]*,tnc,*1_	3.07	–	**Supplementary Material Section 1.2**
*N*_[*ACh*]*,tnc,*3_	2.11	–	**Supplementary Material Section 1.2**
$A_{\left[Ca_{SMC}^{2+}\right],tnc,1}$	23.30	µM	**Supplementary Material Section 1.2**
$A_{\left[Ca_{SMC}^{2+}\right],tnc,3}$	0.71	µM	**Supplementary Material Section 1.2**
$B_{\left[Ca_{SMC}^{2+}\right],tnc,1}$	4.89 × 10^5^	µM	**Supplementary Material Section 1.2**
$B_{\left[Ca_{SMC}^{2+}\right],tnc,3}$	126.17	µM	**Supplementary Material Section 1.2**
$N_{\left[Ca_{SMC}^{2+}\right],tnc,1}$	0.34	–	**Supplementary Material Section 1.2**
$N_{\left[Ca_{SMC}^{2+}\right],tnc,3}$	0.24	–	**Supplementary Material Section 1.2**
*A*_[*VIP*]*,tnc,*1_	67.37	nM	**Supplementary Material Section 1.2**
*B*_[*VIP*]*,tnc,*1_	19.89	Hz	**Supplementary Material Section 1.2**
*N*_[*VIP*]*,tnc,*1_	2.35	–	**Supplementary Material Section 1.2**
*A*_*iψ,*[*VIP*],1_	1	–	**Supplementary Material Section 1.2**
*B*_*iψ,*[*VIP*]1_	1.25	nM	**Supplementary Material Section 1.2**
*N*_*iψ,*[*VIP*],1_	0.82	–	**Supplementary Material Section 1.2**
*A*_[*NO*]*,tnc,*1_	0.68	µM	**Supplementary Material Section 1.2**
*B*_[*NO*]*,tnc,*1_	18	Hz	**Supplementary Material Section 1.2**
*N*_[*NO*]*,tnc,*1_	1.50	–	**Supplementary Material Section 1.2**
*A*_*ih,tnc,*3_	1	–	**Supplementary Material Section 1.2**
*B*_*ih,tnc,*3_	1.03	Hz	**Supplementary Material Section 1.2**
*N*_*ih,tnc,*3_	1.57	–	**Supplementary Material Section 1.2**
*A*_[*ACh*]*,p,*2_	156.43	nM	**Supplementary Material Section 1.2**
*A*_[*ACh*]*,p,*3_	156.43	nM	**Supplementary Material Section 1.2**
*B*_[*ACh*]*,p,*2_	3.54	Hz	**Supplementary Material Section 1.2**
*B*_[*ACh*]*,p,*3_	3.54	Hz	**Supplementary Material Section 1.2**
*N*_[*ACh*]*,p,*2_	3.99	–	**Supplementary Material Section 1.2**
*N*_[*ACh*]*,p,*3_	3.99	–	**Supplementary Material Section 1.2**
*A*_*ea,p,*2_	0.15	–	**Supplementary Material Section 1.2**
*A*_*ea,p,*3_	0.15	–	**Supplementary Material Section 1.2**
*B*_*ea,p,*2_	0.57	nM	**Supplementary Material Section 1.2**
*B*_*ea,p,*3_	0.57	nM	**Supplementary Material Section 1.2**
*N*_*ea,p,*2_	0.33	–	**Supplementary Material Section 1.2**
*N*_*ea,p,*3_	0.33	–	**Supplementary Material Section 1.2**
*A*_*ec,p,*2_	1.3	–	**Supplementary Material Section 1.2**
*A*_*ec,p,*3_	1.3	–	**Supplementary Material Section 1.2**
*B*_*ec,p,*2_	96	nM	**Supplementary Material Section 1.2**
*B*_*ec,p,*3_	96	nM	**Supplementary Material Section 1.2**
*N*_*ec,p,*2_	1	–	**Supplementary Material Section 1.2**
*N*_*ec,p,*3_	1	–	**Supplementary Material Section 1.2**
*A*_[*NO*]*,p,*2_	0.67	µM	**Supplementary Material Section 1.2**
*A*_[*NO*]*,p,*3_	0.67	µM	**Supplementary Material Section 1.2**
*B*_[*NO*]*,p,*2_	43.82	Hz	**Supplementary Material Section 1.2**
*B*_[*NO*]*,p,*3_	43.82	Hz	**Supplementary Material Section 1.2**
*N*_[*NO*]*,p,*2_	1.50	–	**Supplementary Material Section 1.2**
*N*_[*NO*]*,p,*3_	1.50	–	**Supplementary Material Section 1.2**
*A*_*ia,p,*2_	0.38	–	**Supplementary Material Section 1.2**
*A*_*ia,p,*3_	0.38	–	**Supplementary Material Section 1.2**
*B*_*ia,p,*2_	3.79 × 10^−2^	nM	**Supplementary Material Section 1.2**
*B*_*ia,p,*3_	3.79 × 10^−2^	nM	**Supplementary Material Section 1.2**
*N*_*ia,p,*2_	0.75	–	**Supplementary Material Section 1.2**
*N*_*ia,p,*3_	0.75	–	**Supplementary Material Section 1.2**
*A*_*ic,p,*2_	0.12	–	**Supplementary Material Section 1.2**
*A*_*ic,p,*3_	0.12	–	**Supplementary Material Section 1.2**
*B*_*ic,p,*2_	3.53	nM	**Supplementary Material Section 1.2**
*B*_*ic,p,*3_	3.53	nM	**Supplementary Material Section 1.2**
*N*_*ic,p,*2_	1.53	–	**Supplementary Material Section 1.2**
*N*_*ic,p,*3_	1.53	–	**Supplementary Material Section 1.2**
*A*_[*Pur*]*,p,*2_	0.52	µM	**Supplementary Material Section 1.2**
*A*_[*Pur*]*,p,*3_	0.52	µM	**Supplementary Material Section 1.2**
*B*_[*Pur*]*,p,*2_	8.76	Hz	**Supplementary Material Section 1.2**
*B*_[*Pur*]*,p,*3_	8.76	Hz	**Supplementary Material Section 1.2**
*N*_[*Pur*]*,p,*2_	1.74	–	**Supplementary Material Section 1.2**
*N*_[*Pur*]*,p,*3_	1.74	–	**Supplementary Material Section 1.2**
*A*_*iψ,p,*2_	167.59	–	**Supplementary Material Section 1.2**
*A*_*iψ,p,*3_	167.59	–	**Supplementary Material Section 1.2**
*B*_*iψ,p,*2_	907.93	µM	**Supplementary Material Section 1.2**
*B*_*iψ,p,*3_	907.93	µM	**Supplementary Material Section 1.2**
*N*_*iψ,p,*2_	0.84	–	**Supplementary Material Section 1.2**
*N*_*iψ,p,*3_	0.84	–	**Supplementary Material Section 1.2**
*X*_*mech,a*_	42.85	Hz	Section 2.3
*X*_*mech,b*_	−42.85	Hz	Section 2.3
*X*_*chem,a*_	26.39	Hz.s.cm^−3^	Section 2.3
*X*_*chem,b*_	−0.79	Hz	Section 2.3
Ω	1.2	–	Section 2.3
*γ*_*max*_	0.05	kcal.s^−1^	Kong and Singh (2008); Li and Jin (2021)

**Table 2:** Gastric model parameters for simulating the gut–brain axis in gastric function regulation.

Parameter	Value	Unit	Reference
${\left[Ca\right]}_{rest,1}^{2+}$	0.34	µM	**Supplementary Material Section 1.2**
${\left[Ca\right]}_{rest,3}^{2+}$	0.18	µM	**Supplementary Material Section 1.2**
[*cGMP*]*_max_*	26.57	µM	**Supplementary Material Section 1.2.1**
*K_NO_*	21.61	nM	**Supplementary Material Section 1.2.1**
[*MLCP*]_*max,*1_	7.5	µM	**Supplementary Material Section 1.2**
[*MLCP*]_*max,*2_	2.5	µM	**Supplementary Material Section 1.2**
[*MLCP*]_*max,*3_	2.5	µM	**Supplementary Material Section 1.2**
[*MLCP*]*_rest_*	7.5	µM	Gajendiran and Buist (2011)
*N*_[*cGMP*]_	2	–	Yang et al. (2005)
*K*_[*cGMP*]_	5.5	µM	Yang et al. (2005)
*β*_1_	20.69	–	**Supplementary Material Section 1.2**
*β*_3_	236.32	–	**Supplementary Material Section 1.2**
*α*_1_	0.21	–	**Supplementary Material Section 1.2**
*α*_3_	0.13	–	**Supplementary Material Section 1.2**
*κ*_*ini*_	48	mA	Fernandes et al. (2024); Du et al. (2010)
*cpm*_*ini*_	3	cpm	Fernandes et al. (2024); Du et al. (2010)
*G*_*coup*_	1.26	S	Berry et al. (2016); Fernandes et al. (2024)
${\bar{o}}_{A}$	2098.37	kPa	Panda and Buist (2021)
${\bar{o}}_{B}$	72.98	MPa	Panda and Buist (2021)
$\bar{X}$	0.149	–	Panda and Buist (2021)
${\bar{o}}_{opt}$	0.68	–	Panda and Buist (2021)
*S*_3_	4.2	kPa	Fernandes et al. (2024), **Supplementary Material Section 1.2**
*S*_4_	−0.19	–	Fernandes et al. (2024), **Supplementary Material Section 1.2**
*S*_5_	0.04	kPa.s	Fernandes et al. (2024), **Supplementary Material Section 1.2**
*S*_6_	5	kPa^−1^	Fernandes et al. (2024), **Supplementary Material Section 1.2**
*Q*_*max,flow*_	1.67	cm^3^.s^−1^	**Supplementary Material Section 1.2.3**
*r*_*max,*3_	0.48	cm	**Supplementary Material Section 1.2.3**

At the neural level, vagal efferent firing represent motor commands from brainstem and enteric circuits. Neurotransmitter release at the neuromuscular junction is modeled using Michaelis-Menten equation with a Hill coefficient (MMEHC) equation, capturing saturation and nonlinear firing activity. The same formulation is used for receptor-mediated signaling in SMC and ICC, linking neural activity to intracellular biochemical responses.

At the cellular level, neurotransmitter concentrations regulate intracellular calcium dynamics and kinase–phosphatase balance. In tonic regions such as the fundus, cholinergic signaling increases intracellular calcium to promote contraction, while inhibitory NANC signaling modulates myosin light chain kinase (MLCK) and myosin light chain phosphatase (MLCP) activity to promote relaxation. In phasic regions such as the antrum and PS, excitatory and inhibitory inputs regulate ICC slow-wave amplitude and frequency, thereby controlling the timing and strength of peristaltic contractions.

At the tissue mechanics level, intracellular signaling is translated into smooth muscle contraction through actin–myosin cross- and latch-bridge kinetics governed by the Hai–Murphy model. Active and passive stresses jointly determine tissue deformation and stretch, enabling tonic relaxation, phasic occlusion, and coordinated gastric contractions.

At the organ level, the stomach compartments remain mechanically coupled through shared volume and stress relationships. Changes in smooth muscle activity alter compartmental radius, which directly governs gastric flow. Gastric emptying through the PS is determined by its instantaneous radius using a simplified flow relationship.

**Figure 1:**
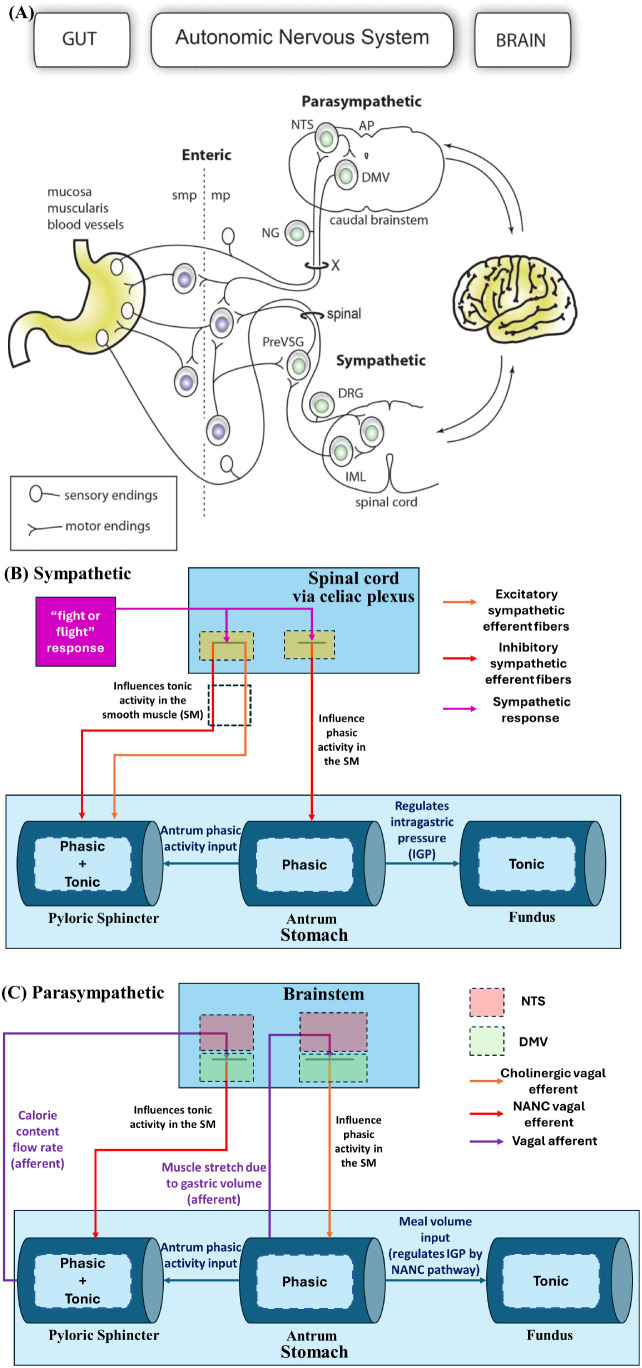
**(A)** Gut-brain axis schematic: sympathetic and parasympathetic pathways for gastric function regulation by (Udit and Gautron, 2013). Purple circles represent enteric nervous system (ENS) neurons, green circles represent extrinsic autonomic nervous system (ANS) neurons **(B)** Compartmental model schematic for sympathetic pathway **(C)** Compartmental model schematic for parasympathetic pathway for gastric function regulation during gastric emptying.

Finally, sensory feedback closes the gut–brain loop. Mechanosensitive afferents encode gastric wall stretch via the fundic deformation parameter, while chemosensitive afferents encode nutrient flow into the duodenum. These afferent signals are transformed into efferent motor output through fitted interneuronal transfer functions representing brainstem processing, enabling adaptive regulation of gastric motility. A schematic overview of the integrated sympathetic and parasympathetic pathways considered in the framework is provided in **Figure 1**.

### 2.2 Modeling the efferent/motor neuron connections and the stomach compartments

Literature reports (Kindt and Tack, 2006; Oberhelman and H., 1966; Armitage and Dean, 1963; Lu et al., 2022; Goyal et al., 2019) indicate that gastric function varies significantly across three primary regions of the stomach: the fundus, antrum, and PS. This regional specialization motivates the use of a three-compartment model in the present study.

Such a representation also enables differentiation of motor neuron targets according to the functional role of each gastric region. In particular, contraction patterns differ across these regions: the fundus primarily exhibits tonic activity, the antrum displays phasic activity, and the PS exhibits both tonic and phasic activity (Zhang et al., 2013; Lüdtke et al., 1991; Friedenberg et al., 2008; Ruckebusch and Malbert, 1986).

In the subsequent subsections, motor neuron activity—both inhibitory and excitatory—will be discussed, and mathematical formulations for these activities will be developed for each of these stomach regions.

#### 2.2.1 Motor neurons influencing tonic activity: fundus

Unlike other regions of the stomach, the fundus (or proximal stomach) lacks ICCs, which are essential to generate phasic contractions. Consequently, the fundus primarily maintains basal tone (Du et al., 2010; Zhang et al., 2013). The NANC inhibitory pathway reduces basal tone and serves as a critical mechanism for fundic relaxation (Curro et al., 2008). In contrast, the cholinergic excitatory pathway increases basal tone and facilitates fundic contractions (Azpiroz and Malagelada, 1987).

The NANC inhibitory pathway mediates fundic relaxation through the release of nitric oxide (NO) and vasoactive intestinal peptide (VIP) as primary neurotransmitters. At low neuronal firing frequencies, NO is released at the neuromuscular junction to induce relaxation (Currò and Preziosi, 1998; D’Amato et al., 1992; Boeckxstaens et al., 1992). At higher neuronal firing frequencies, NO and VIP are released simultaneously, further enhancing the relaxation response (Currò et al., 2002).

**Figure 2:**
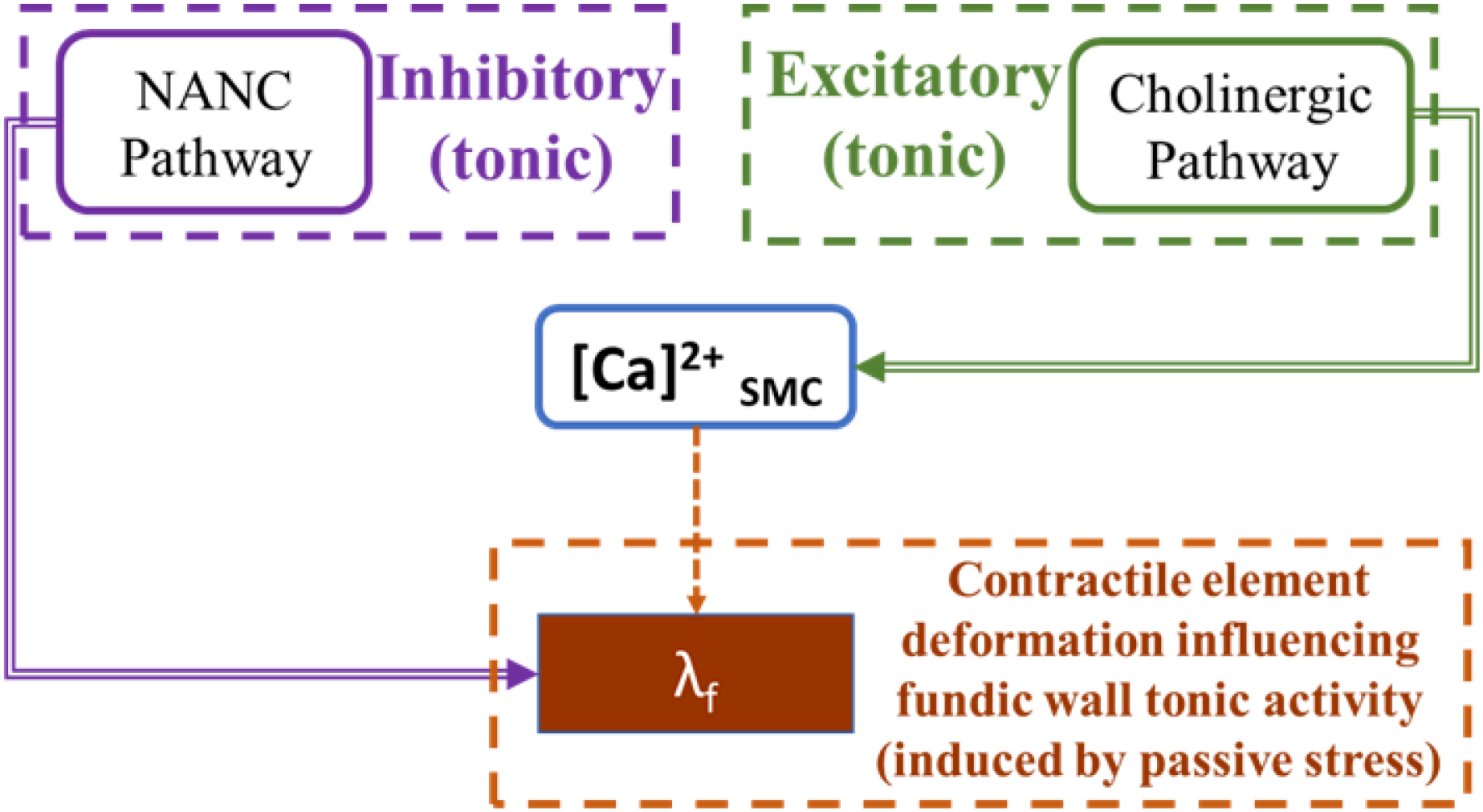
Schematic representation of excitatory and inhibitory inputs to the fundic tonic activity. The excitatory cholinergic pathway modulates intracellular calcium concentration in smooth muscle cells, $\left[Ca_{SMC}^{2+}\right]$, whereas the inhibitory non-adrenergic non-cholinergic (NANC) pathway affects the contractileelement deformation parameter *λ_f_* through inhibitory signaling mechanisms. These pathways together govern tonic fundic wall activity associated with passive stress.

The cholinergic excitatory pathway induces the release of acetylcholine (ACh) at the neuromuscular junction, promoting an increase in fundic contractions (Kim et al., 2020). The combined effects of these excitatory and inhibitory efferent neural pathways influence deformation of the contractile element through the passive stretch variable *λ_f_*, which in turn modulates tonic contraction in the fundus. A schematic representation of the excitatory and inhibitory pathways in the fundus (*w* = 1) is shown in **Figure 2**.

To model the relationship between motor neuron firing frequency and neurotransmitter release at the neuromuscular junction, MMEHC equation is employed. This equation effectively models the trends reported in a previous study on neuron firing and neurotransmitter release at the neuromuscular junction (Briant et al., 2015).

The inclusion of the Hill coefficient allows the equation to account for the nonlinear behavior often observed in neurotransmitter release dynamics. Additionally, the Michaelis-Menten formulation incorporates a saturation term, which is critical for representing the physiological limit of neurotransmitter release.

For simulating neurotransmitter receptor signaling in smooth muscles, the MMEHC equation is equally suitable. It is widely used to model phenomenological ligand-receptor interactions and is therefore employed to describe both neurotransmitter release and subsequent receptor-mediated signaling behaviors (Mercer, 2023; Gesztelyi et al., 2012). Further details on the application of the MMEHC can be found in the **Supplementary Materials Section 1.1**.

The general form of the MMEHC, given in Equation 1, is as follows:

(1) $$P_{ℳ}=\frac{A_{ℳ}X_{O}^{N_{ℳ}}}{B_{ℳ}^{N_{ℳ}}+X_{O}^{N_{ℳ}}}.$$

In this equation, *A* denotes the maximum response, *B* is the Michaelis constant, and *N* is the Hill coefficient. The subscript ℳ refers to the modality of the interaction, such as neurotransmitter type, inhibitory or excitatory activity, tonic (*to*) or phasic (*p*) contraction type, or compartment index *w*. The variable X represents the input signal, which may correspond to a neurotransmitter concentration, a signaling molecule concentration, or the firing frequency of excitatory or inhibitory neurons. The subscript O designates the origin or context of this input, including notations such as *e* (excitatory), *i* (inhibitory), *to* (tonic), *p* (phasic), or compartment index *w*.

In this study, multiple equations—specifically Equations 2–6, 8, 9, 17–24 and 45 are formulated based on the generalized MMEHC expression and follow a similar form, with the respective subscripts.

#### 2.2.2 Cholinergic pathway (ACh): fundus

In the fundus, the neurotransmitter ACh is released at the neuromuscular junction via the cholinergic pathway (Azpiroz and Malagelada, 1987). ACh increases intracellular calcium concentration ${\left[Ca_{SMC}^{2+}\right]}_{tnc,1}$ in the smooth muscle, influencing fundic contractions (Briant et al., 2015). Accordingly, the concentration of *ACh* released is modeled as a function of the cholinergic pathway firing frequency *f_e,tnc,_*_1_. This relationship is expressed as follows.

(2) $${\left[ACh\right]}_{1}=\frac{A_{\left[ACh\right],tnc,1}f_{e,tnc,1}^{N_{\left[ACh\right],tnc,1}}}{B_{\left[ACh\right],tnc,1}^{N_{\left[ACh\right],tnc,1}}+f_{e,tnc,1}^{N_{\left[ACh\right],tnc,1}}}$$

The intracellular calcium concentration in the SMC, ${\left[Ca_{SMC}^{2+}\right]}_{tnc,1}$, influenced by tonic cholinergic neurotransmitter signaling is modeled by the following equation.

(3) $${\left[Ca_{SMC}^{2+}\right]}_{tnc,1}=\frac{A_{\left[Ca_{SMC}^{2+}\right],tnc,1}{\left[ACh\right]}_{1}^{N_{\left[Ca_{SMC}^{2+}\right],tnc,1}}}{B_{\left[Ca_{SMC}^{2+}\right],tnc,1}^{N_{\left[Ca_{SMC}^{2+}\right],tnc,1}}+{\left[ACh\right]}_{1}^{N_{\left[Ca_{SMC}^{2+}\right],tnc,1}}}$$

Data from (Kim et al., 2020; da Silva et al., 2018) were used to estimate the parameters of the cholinergic signaling response for Equations 2 and 3. This calcium concentration in turn affects the contractile element stress *λ_f_* and fundus tonic activity which we discuss in later sections.

#### 2.2.3 NANC pathway (NO and VIP): fundus

The neurotransmitters NO and VIP are released at the neuromuscular junction of the fundus via NANC pathway signaling (Tonini et al., 2000). Based on the firing frequency of the NANC pathway *f_i,tnc,_*_1_, the concentration of the neurotransmitter VIP released at the neuromuscular junction is described by the MMEHC, represented in Equation 4, as follows.

(4) $${\left[VIP\right]}_{1}=\frac{A_{\left[VIP\right],tnc,1}f_{i,tnc,1}^{N_{\left[VIP\right],tnc,1}}}{B_{\left[VIP\right],tnc,1}^{N_{\left[VIP\right],tnc,1}}+f_{i,tnc,1}^{N_{\left[VIP\right],tnc,1}}}$$

The VIP neurotransmitter is known to inhibit MLCK activity via the cyclic adenosine monophosphate (cAMP) signaling pathway (Yang et al., 2005; Shiraga et al., 1994). In the present model, this effect is represented through a reduction in the effective activated MLCK activity. The MLCK activity inhibitory factor *ϕ_iψ,_*_1_ is modeled by Equation 5 as follows.

(5) $$\phi_{i\psi ,1}=\frac{A_{i\psi ,tnc,1}{\left[VIP\right]}_{1}^{N_{i\psi ,tnc,1}}}{B_{i\psi ,tnc,1}^{N_{i\psi ,tnc,1}}+{\left[VIP\right]}_{1}^{N_{i\psi ,tnc,1}}}$$

Based on the firing frequency *f_i,tnc,_*_1_, the release of the neurotransmitter NO at the neuromuscular junction is represented by the following equation.

(6) $${\left[NO\right]}_{1}=\frac{A_{\left[NO\right],tnc,1}f_{i,tnc,1}^{N_{\left[NO\right],tnc,1}}}{B_{\left[NO\right],tnc,1}^{N_{\left[NO\right],tnc,1}}+f_{i,tnc,1}^{N_{\left[NO\right],tnc,1}}}$$

Data from previous studies (Hayes et al., 1999; Takahashi and Owyang, 1995; Jenkinson and Reid, 2000; Curro et al., 2008) were used to estimate the NO-mediated neurotransmitter inhibitory response through increased MLCP concentration, which will be discussed in the following section. Additionally, data from (Hayes et al., 1999; Costa et al., 1986; Curro et al., 2008; Takahashi and Owyang, 1995; Grider et al., 1985) were used to fit the inhibitory response associated with the reduction in MLCK activity.

#### 2.2.4 Equations in the compartmental model for fundus

The total intracellular calcium concentration in the fundus SMC ${\left[Ca\right]}_{SMC,1}^{2+}$ is the sum of the resting calcium concentration ${\left[Ca\right]}_{rest,1}^{2+}$ in the fundus and the increase in calcium concentration influenced by cholinergic signaling ${\left[Ca_{SMC}^{2+}\right]}_{tnc,1}$. The relationship represented by Equation 7 is expressed as.

(7) $${\left[Ca\right]}_{SMC,1}^{2+}={\left[Ca\right]}_{rest,1}^{2+}+{\left[Ca_{SMC}^{2+}\right]}_{tnc,1}$$

The model from (Gajendiran and Buist, 2011) is employed to describe the kinetics that convert intracellular calcium concentration into active MLCK concentration [*MLCK_act_*]*_tnc,_*_1_, which serves as a proxy for MLCK activity.

NO signaling increases MLCP concentration via the cyclic guanosine monophosphate (cGMP) pathway, as demonstrated in studies by (Nakamura et al., 2007; Yang et al., 2005). Data from (Yang et al., 2005) were used to fit a Michaelis-Menten kinetic equation modeling the relationship between NO and cGMP.

The cGMP concentration [*cGMP*]*_tnc,_*_1_ in the fundus is represented as.

(8) $${\left[cGMP\right]}_{tnc,1}=\frac{[cGMP{]}_{max}[NO{]}_{1}}{K_{\left[NO\right]}+{\left[NO\right]}_{1}}$$

The model presented in (Yang et al., 2005) was applied to describe the relationship between cGMP and MLCP. The increase in MLCP concentration [*MLCP*]*_f,tnc,_*_1_ in the fundus is described by the following equation.

(9) $${\left[MLCP\right]}_{f,tnc,1}=\frac{{\left[MLCP\right]}_{max,1}{\left[cGMP\right]}_{tnc,1}^{N_{\left[cGMP\right]}}}{K_{\left[cGMP\right]}^{N_{\left[cGMP\right]}}+{\left[cGMP\right]}_{tnc,1}^{N_{\left[cGMP\right]}}}$$

The total MLCP concentration is given as ${\left[MLCP\right]}_{tnc,1}={\left[MLCP\right]}_{rest}+{\left[MLCP\right]}_{f,tnc,1}$. Similarly, the total activated MLCK concentration is expressed as ${\left[MLCK\right]}_{tnc,1}=\phi_{i\psi ,1}{\left[MLCK_{act}\right]}_{tnc,1}$, where *ϕ_iψ,_*_1_ represents the inhibitory factor induced by VIP neurotransmitter signaling and thereby reduces effective MLCK activity.

The Hai-Murphy model (Hai and Murphy, 1988), as reported in (Gajendiran and Buist, 2011), is utilized to compute the total number of latch bridges formed. Tonic contractions, being sustained contractions, result in the formation of latch bridges as cross-bridge states which transition into latch bridges during sustained contractions (Murphy and Rembold, 2005).

The total number of latch bridges is represented as the sum of ${\left[AM_{p}\right]}_{tnc,1}$ and ${\left[AM\right]}_{tnc,1}$. The Equations 10-14 for modeling the formulation of latch bridges based on the Hai-Murphy model are given as follows.

(10) $$\frac{d{\left[M\right]}_{tnc,1}}{dt}=-\frac{k_{cat,MLCK_{act}}{\left[MLCK\right]}_{tnc,1}{\left[M\right]}_{tnc,1}}{k_{m,MLCK_{act}}+{\left[M\right]}_{tnc,1}}+\frac{k_{cat,MLCP}{\left[MLCP\right]}_{tnc,1}{\left[M_{p}\right]}_{tnc,1}}{k_{m,MLCP}+{\left[M_{p}\right]}_{tnc,1}}+k_{a}{\left[AM\right]}_{tnc,1}$$

(11) $$\frac{d{\left[M_{p}\right]}_{tnc,1}}{dt}=\frac{k_{cat,MLCK_{act}}[MLCK{]}_{tnc,1}[M{]}_{tnc,1}}{k_{m,MLCK_{act}}+{\left[M\right]}_{tnc,1}}-\frac{k_{cat,MLCP}[MLCP{]}_{tnc,1}[M_{p}{]}_{tnc,1}}{k_{m,MLCP}+{\left[M_{p}\right]}_{tnc,1}}-k_{b}[M_{p}{]}_{tnc,1}+k_{c}[AM_{p}{]}_{tnc,1}$$

(12) $$\frac{d{\left[AM_{p}\right]}_{tnc,1}}{dt}=k_{b}{\left[M\right]}_{tnc,1}-k_{c}{\left[AM_{p}\right]}_{tnc,1}+\frac{k_{cat,MLCK_{act}}{\left[MLCK\right]}_{tnc,1}{\left[AM\right]}_{tnc,1}}{k_{m,MLCK_{act}}+{\left[AM\right]}_{tnc,1}}-\frac{k_{cat,MLCP}{\left[MLCP\right]}_{tnc,1}{\left[AM_{p}\right]}_{tnc,1}}{k_{m,MLCP}+{\left[AM_{p}\right]}_{tnc,1}}$$

(13) $$\frac{d{\left[AM\right]}_{tnc,1}}{dt}=-\frac{k_{cat,MLCK_{act}}{\left[MLCK\right]}_{tnc,1}{\left[AM\right]}_{tnc,1}}{k_{m,MLCK_{act}}+{\left[AM\right]}_{tnc,1}}+\frac{k_{cat,MLCP}{\left[MLCP\right]}_{tnc,1}]{\left[M_{p}\right]}_{tnc,1}}{k_{m,MLCP}+{\left[AM_{p}\right]}_{tnc,1}}-k_{a}{\left[AM\right]}_{tnc,1}$$

The relative area for the distension of the stomach in the fundus compartment *RA*_1_ as a function of the total number of latch bridges is calculated using Equation 14 reported in (Wang et al., 2008), expressed as.

(14) $$RA_{1}={\left(\frac{1-\beta_{1}\left({\left[AM\right]}_{tnc,1}+{\left[AM_{p}\right]}_{tnc,1}\right)}{1-\beta_{1}\left({\left[AM\right]}_{max,tnc,1}+{\left[AM_{p}\right]}_{max,tnc,1}\right)}\right)}^{2}$$

Here *β*_1_ is a dimensionless parameter. Considering the stomach can be approximated as a cylinder (Fernandes et al., 2024), the radius *r_fin_* of the fundus compartment is computed using the open cylinder formulation, given in Equation 15 as.

(15) $$r_{fin,1}=RA_{1}r_{ini,1}$$

The radius of the open cylinder fundus at its minimum volume, *r_ini,_*_1_, corresponds to the unstressed radius of the stomach, calculated when the gastric volume is at a minimum of 0.08 L.

The contractile element deformation of the muscle fiber *λ_f,_*_1_, which influences the passive stress in the fundus wall, is determined by a linear relationship involving the ratio of the stressed radius to the unstressed radius, as defined in the study by (Pironet et al., 2013). The dimensionless constant *α*_1_ is introduced to fit *λ_f,_*_1_ within the physiological range.

The equation for the passive stress contractile element deformation *λ_f,_*_1_ described by Equation 16, is expressed as.

(16) $$\lambda_{f,1}=\alpha_{1}\left(\frac{r_{fin,1}}{r_{ini,1}}-1\right)+1$$

#### 2.2.5 Motor neurons influencing phasic activity: antrum

The antrum of the stomach plays a pivotal role in mixing and grinding ingested food (Fernandes et al., 2024). It is densely populated with ICCs, which generate the slow-wave electrical activity responsible for initiating the phasic contractions characteristic of this region. These contractions occur in a coordinated process known as peristalsis, propelling food toward the pylorus (Du et al., 2010; Fernandes et al., 2024; Ferrua and Singh, 2010; Li and Jin, 2021).

Phasic contractions of gastric smooth muscle are driven by neural inputs from the vagal nerve and the ENS, which target the ICC and SMC within the gastric wall (Travagli et al., 2006; Athavale et al., 2024a; Beckett et al., 2017). As demonstrated in the study by (Athavale et al., 2024a), these contractions are regulated by both cholinergic (excitatory) and NANC (inhibitory) pathways. The cholinergic pathway enhances contractile behavior, whereas inhibitory mechanisms—subdivided into purinergic and nitrergic pathways—reduce contraction activity. Together, these pathways modulate the balance of excitatory and inhibitory inputs, finely tuning gastric motility within the antrum (Athavale et al., 2024a; Travagli and Anselmi, 2016; Forrest et al., 2006; Kim et al., 2003).

**Figure 3:**
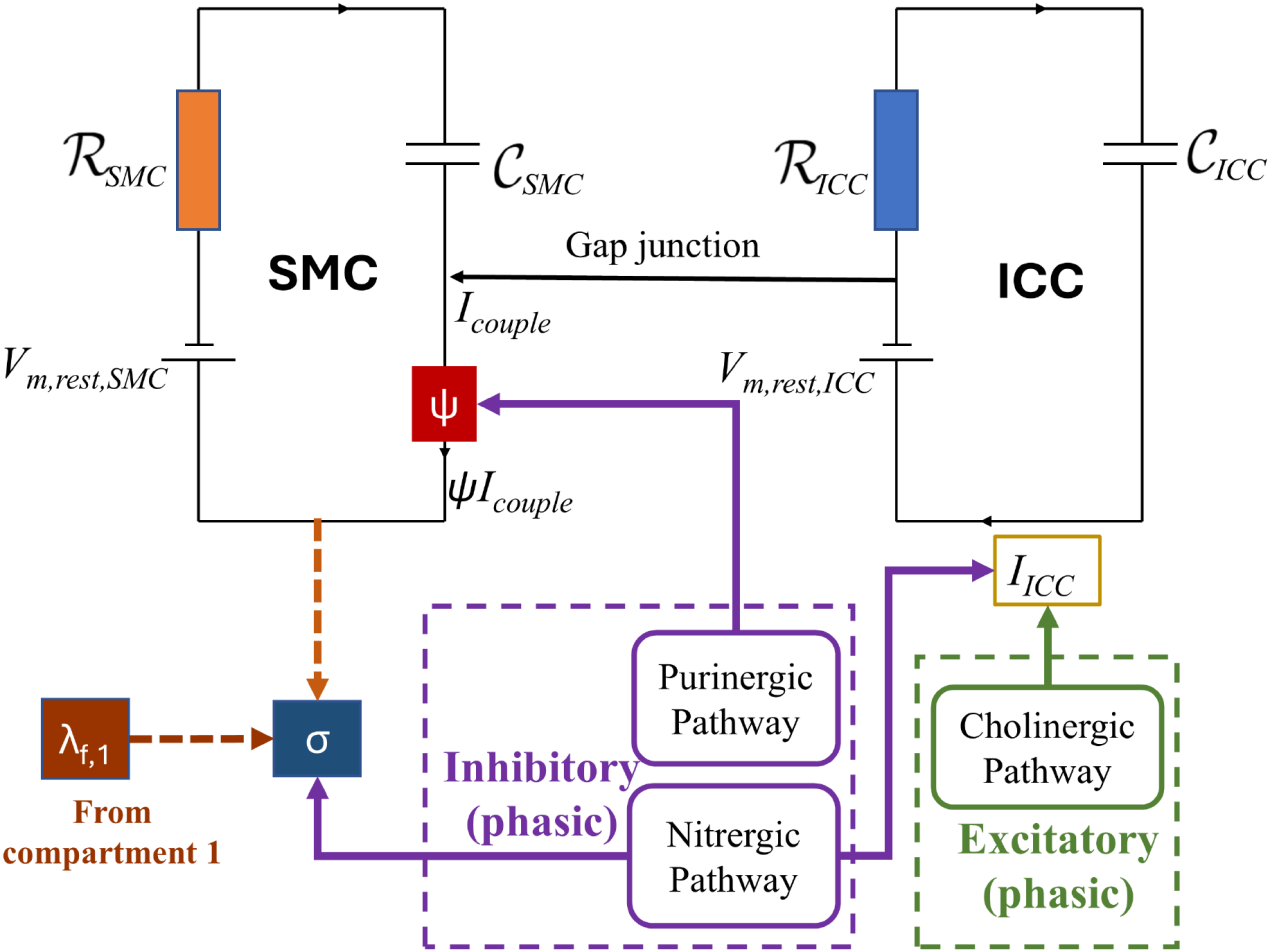
Schematic representation of excitatory and inhibitory stimulation for antrum phasic activity. Smooth muscle cells (SMC) and interstitial cells of Cajal (ICC) are modeled as electrically coupled compartments, with coupling current *I_couple_* flowing through gap junctions. The excitatory cholinergic pathway modulates ICC activity through *I_ICC_*, whereas the inhibitory purinergic and nitrergic pathways influence both ICC and SMC activity. Inhibitory signaling also affects tissue stress σ, which modulates smooth muscle behavior together with the fundic input μ*_f_*_,1_ from compartment 1. Here, ℛ denotes membrane resistance, C denotes membrane capacitance, $V_{m,rest,SMC}$ and $V_{m,rest,ICC}$ denote the resting membrane potentials of SMC and ICC, respectively, and $\psi I_{couple}$ denotes the inhibitory-response modulation term associated with the gap-junction coupling current.

For modeling the motor neuron firing frequency of both excitatory and inhibitory pathways, the MMEHC is employed, following a similar methodology as discussed in Section 2.2.1. This modeling approach is integrated into the compartmental framework (Fernandes et al., 2024), extending the modified ‘leaky integrate and fire’ (MLIF) model by incorporating excitatory and inhibitory neural inputs modeled by the MMEHC for the antrum region (*w* = 2) of the stomach. A schematic representation of this framework is provided in **Figure 3**.

#### 2.2.6 Cholinergic pathway (ACh): antrum

In the antrum, the cholinergic pathway is regulated by the neurotransmitter ACh, which is released at the neuromuscular junction. The ICC responds to this neurotransmitter, eliciting an excitatory effect on both the amplitude and frequency of the ICC phasic slow waves that are essential for coordinated motility (Athavale et al., 2024a; Nakamura and Suzuki, 2004).

The release of ACh [*ACh*]_2_ in response to the firing frequency of the cholinergic pathway *f_e,p,_*_2_ is modeled by Equation 17 as follows.

(17) $${\left[ACh\right]}_{2}=\frac{A_{\left[ACh\right],p,2}f_{e,p,2}^{N_{\left[ACh\right],p,2}}}{B_{\left[ACh\right],p,2}^{N_{\left[ACh\right],p,2}}+f_{e,p,2}^{N_{\left[ACh\right],p,2}}}$$

The fractional increase in the ICC slow-wave amplitude, *ϕ_ea,_*_2_, in response to the ACh concentration [*ACh*]_2_, is modeled by Equation 18 as follows.

(18) $$\phi_{ea,2}=\frac{A_{ea,p,2}{\left[ACh\right]}_{2}^{N_{ea,p,2}}}{B_{ea,p,2}^{N_{ea,p,2}}+{\left[ACh\right]}_{2}^{N_{ea,p,2}}}$$

The fractional increase of ICC slow-wave frequency, $\phi_{ea,2}$, in response to ACh concentration is expressed by Equation 19 as follows equation.

(19) $$\phi_{ca,2}=\frac{A_{ec,p,2}{\left[ACh\right]}_{2}^{N_{ec,p,2}}}{B_{ec,p,2}^{N_{ec,p,2}}+{\left[ACh\right]}_{2}^{N_{ec,p,2}}}$$

Data from studies in (Sinn et al., 2010; Forrest et al., 2006) was utilized to estimate the parameters of the MMEHC equations describing the relationship between cholinergic pathway firing frequency and ICC slow-wave frequency. Additionally, data from (Athavale et al., 2024a; Nakamura and Suzuki, 2004) was employed to determine the parameters of the MMEHC equations modeling the increase in ICC slow-wave amplitude as a response to cholinergic pathway firing frequency.

#### 2.2.7 NANC pathway (NO and purinergic): antrum

The inhibitory response in the antrum is modulated by the NANC pathway, which includes nitrergic neurotransmitters like NO and purinergic neurotransmitters like adenosine triphosphate (ATP), released at the neuromuscular junction (Athavale et al., 2024a; Burnstock, 2006; Beck et al., 2018). These neurotransmitters act on SMCs and ICCs to reduce the amplitude and frequency of contractions, thereby playing a vital role in balancing excitatory and inhibitory inputs within the gastric motility system.

To model nitrergic neurotransmitter release in ICC, the concentration of NO neurotransmitter, ${\left[NO\right]}_{p,2}$, at the neuromuscular junction is modeled by Equation 20 as a function of the NANC firing frequency *f_i_*,*_p_*_,2_, which influences inhibitory signaling and ICC activity. The equation is formulated as follows:

(20) $${\left[NO\right]}_{2}=\frac{A_{\left[NO\right],p,2}f_{i,p,2}^{N_{\left[NO\right],p,2}}}{B_{\left[NO\right],p,2}^{N_{\left[NO\right],p,2}}+f_{i,p,2}^{N_{\left[NO\right],p,2}}}$$

The fractional reduction in ICC slow-wave amplitude, $\phi_{ia,2}$, based on NO concentration, is represented by Equation 21 as the following.

(21) $$\phi_{ia,2}=\frac{A_{ia,p,2}{\left[NO\right]}_{2}^{N_{ia,p,2}}}{B_{ia,p,2}^{N_{ia,p,2}}+{\left[NO\right]}_{2}^{N_{ia,p,2}}}$$

The fractional reduction in ICC slow-wave frequency, *ϕ_ic,_*_2_, based on the NO concentration, is denoted Equation 22 as follows.

(22) $$\phi_{ic,2}=\frac{A_{ic,p,2}{\left[NO\right]}_{2}^{N_{ic,p,2}}}{B_{ic,p,2}^{N_{ic,p,2}}+{\left[NO\right]}_{2}^{N_{ic,p,2}}}$$

To model purinergic neurotransmitter release in SMC, the concentration of purinergic neurotransmitter [*Pur*]_2_ at the neuromuscular junction is modeled as a function of the efferent firing frequency *f_i,p,_*_2_, which influences neurotransmitter availability and the subsequent smooth muscle response. The equation is represented as Equation 23 as follows.

(23) $${\left[Pur\right]}_{2}=\frac{A_{\left[Pur\right],p,2}f_{i,p,2}^{N_{\left[Pur\right],p,2}}}{B_{\left[Pur\right],p,2}^{N_{\left[Pur\right],p,2}}+f_{i,p,2}^{N_{\left[Pur\right],p,2}}}$$

The purinergic neurotransmitter at the neuromuscular junction modulates the SMC slow-wave amplitude (Athavale et al., 2024a). The fractional reduction in the SMC slow-wave amplitude, denoted by $\phi_{i\psi ,2}$, in response to purinergic neurotransmitter concentration which ios modeled by Equation 24 as follows.

(24) $$\phi_{i\psi ,2}=\frac{A_{i\psi ,p,2}{\left[Pur\right]}_{2}^{N_{i\psi ,p,2}}}{B_{i\psi ,p,2}^{N_{i\psi ,p,2}}+{\left[Pur\right]}_{2}^{N_{i\psi ,p,2}}}$$

The inhibitory fractional response to the purinergic neurotransmitter, denoted as *ψ*_2_, is represented Equation 25 as follows.

(25) $$\psi_{2}=\left(1-\phi_{i\psi ,2}\right)$$

#### 2.2.8 Equations in the compartmental model for the gastric antrum

The equation for the ICC active stimulating current amplitude, which incorporates both the fractional excitatory and inhibitory responses, is denoted by Equation 26 as follows.

(26) $$\kappa_{2}=\kappa_{ini}\left(\phi_{ea,2}-\phi_{ia,2}+1\right)$$

Here, *κ_ini_* represents the baseline active stimulating current amplitude. The equation for the ICC slow-wave frequency, which incorporates both the fractional excitatory and inhibitory responses, is denoted by Equation 27 as follows.

(27) $$cpm_{2}=cpm_{ini}\left(\phi_{ec,2}-\phi_{ic,2}+1\right)$$

Here, *cpm_ini_* represents the baseline ICC slow-wave frequency.

The equation for the parameter in the MLIF model that influences the time for each wave cycle, *t_end,_*_2_, is denoted by Equation 28 as follows.

(28) $$t_{end,2}=\frac{60}{cpm_{2}}$$

More details on *κ_m_* and *t_end,_*_2_ can be found in our previous study (Fernandes et al., 2024).

For the ICC, the slow-wave activity is represented by the MLIF model (Fernandes et al., 2024) by Equation 29 as.

(29) $$\frac{dV_{m,ICC,2}}{dt}=\frac{I_{m}{ℛ}_{ICC}-\left(V_{m,ICC,2}-V_{mrest,ICC}\right)}{\tau_{m,ICC}}$$

where, denoted by Equation 30 is represented as.

(30) $$I_{2}=\{\begin{array}{ll} \kappa_{2}, & \text{if }t_{cyc}\leq t_{open}.\\ 0, & \text{otherwise} \end{array}$$

A gap junction equation connects the ICC and SMC models which is denoted by Equation 31 as.

(31) $$I_{couple,2}=G_{coup}\left(V_{m,SMC,2}-V_{m,ICC,2}\right)$$

For the SMC slow-wave activity is modeled (Fernandes et al., 2024) by Equation 32 as.

(32) $$\frac{dV_{m,SMC,2}}{dt}=\frac{\psi I_{couple,2}{ℛ}_{SMC}-\left(V_{m,SMC,2}-V_{mrest,SMC}\right)}{\tau_{m,SMC}}$$

The translation of SMC membrane voltage, *V_m,SMC,_*_2_, to activated MLCK, [*MLCK*]*_p,_*_2_, is based on the framework introduced in our previous work (Fernandes et al., 2024). This framework builds upon models developed by (Corrias and Buist, 2007; Gajendiran and Buist, 2011).

The total MLCP concentration, [*MLCP*]*_p,_*_2_, is expressed by Equation 33 as.

(33) $$[MLCP{]}_{p,2}=[MLCP{]}_{rest}+{\left[MLCP\right]}_{f,p,2}$$

where [*MLCP*]*_f,p,_*_2_ is computed using Equations 8 and 9.

The interplay between [*MLCP*]*_p,_*_2_ and [*MLCK*]*_p,_*_2_ regulates the number of total cross-bridges formed ([*AM_p_*]*_p,_*_2_ + [*AM*]*_p,_*_2_), which directly influence active muscle contractions (Pollack, 1983; Huxley, 1969). The formation of cross-bridges is modeled using the Hai-Murphy framework, as represented by Equations 10–13.

The tissue stress, *σ*_2_, in the antrum is influenced by contractile element deformation in the fundus compartment, denoted as *λ_f,_*_1_. This deformation reflects the passive distension of the stomach, which may result from the gastric volume of liquid and the total number of cross-bridges formed (Panda and Buist, 2021). The muscle tissue stress *σ*_2_ is modeled using a framework established in previous studies (Murtada et al., 2012; Murtada and Holzapfel, 2014; Panda and Buist, 2021). The governing equations for this relationship are given in Equations 34–37 as follows.

(34) $${\bar{o}}_{mech,2}=\lambda_{f,1}-1$$

(35) $${\bar{o}}_{chem}=-\frac{{\bar{o}}_{A}}{{\bar{o}}_{B}}$$

(36) $${\bar{o}}_{2}={\bar{o}}_{mech,2}+{\bar{o}}_{chem}$$

(37) $${\bar{L}}_{2}={\bar{o}}_{2}-\frac{{\bar{o}}_{2}^{2}}{2{\bar{o}}_{opt}}+\bar{X}$$

Here, ${\bar{o}}_{mech,2}$ represents the sliding filament component caused by passive deformation, while ${\bar{o}}_{chem,2}$ corresponds to the sliding element resulting from active cross-bridging. The normalized actin–myosin filament overlap length is denoted as $\bar{L}$, and the normalized initial actin–myosin filament overlap length is represented as $\bar{X}$. Since the stomach empties gradually rather than instantaneously, the components controlling the stress in the stomach tissue are assumed to be in a steady state. This assumption ensures that the internal and active stresses remain equal at all times. Under this condition, *σ*_2_ is expressed by Equation 38 as follows (Murtada et al., 2012; Panda and Buist, 2021).

(38) $$\sigma_{2}={\bar{o}}_{B}{\bar{L}}_{2}\left([AM_{p}{]}_{p,2}+[AM{]}_{p,2}\right)\left(\lambda_{f,1}-{\bar{o}}_{2}-1\right)$$

The non-linear viscoelastic model (NLVM) model is used to translate tissue stress into tissue stretch, following a framework similar to that of our previous study (Fernandes et al., 2024). However, for the principal stress *E* of the hyperelastic tissue model, a polynomial equation is employed to represent the hyperelastic material, as demonstrated in the study by (Panda and Buist, 2018). For further details, refer to **Supplementary Materials Section 1.2.2**. The principal stress *E*_2_ is expressed by Equation 39 as follows.

(39) $$E_{2}=\sum_{s=1}^{5}A_{s-1}\lambda_{2}^{s}$$

The polynomial coefficients used to represent the principal stress are denoted by $A_{s-1}$. The viscoelastic behavior, including the hysteresis loop, is captured using a nonlinear dashpot model, *η*_2,2_. The parameters *S*_3_, *S*_4_, *S*_5_, and *S*_6_ re fitting constants of this nonlinear dashpot model, similar to the approach described in our previous work (Fernandes et al., 2024). The tissue stretch *λ*_2_ resulting from tissue stress is expressed by Equation 40 as.

(40) $$\frac{d\lambda_{2}}{dt}=\frac{\sigma_{2}-E_{2}}{\eta_{2,2}}$$

where the non-linear dashpot of the model is given by Equations 41, 42 as.

(41) $$\eta_{2,2}=\eta_{1,2}\tanh\;\left(S_{6}{\left[\lambda_{2}-0.98\right]}^{2}\right)$$

(42) $$\eta_{1,2}=S_{3}e^{S_{4}\left(S_{5}E_{2}-\sigma_{2}\right)}$$

The radius of the cylindrical compartment, *r_fin_*_,2_, modeled using the framework from our previous study (Fernandes et al., 2024), is expressed by Equation 43 as.

(43) $$r_{fin,2}=r_{ini,2}-r_{d,2}$$

where.

(44) $$r_{d,2}=\sqrt{\omega_{3,2}^{2}-\frac{L_{t,2}^{2}}{4}}$$

Here, *r_d_*_,2_ denoted by Equation 44, represents the deformed compartmental radius, *L_t_*_,2_ denotes the undeformed tissue length, and *ω*_3,2_ is the component that accounts for the deformed tissue length.

#### 2.2.9 Motor neurons influencing tonic and phasic activity: PS

The PS (*w* = 3) regulates gastric flow from the stomach to the duodenum. Vagal regulation of the PS involves both cholinergic and NANC pathways, which are primarily responsible for maintaining the basal tone of the sphincter through tonic contractions (Richardson et al., 2023). The cholinergic pathway increases basal tone by inducing smooth muscle contraction, while the NANC pathway reduces basal tone by promoting smooth muscle relaxation. ACh is likely the primary neurotransmitter for smooth muscle contraction in the sphincter. However, the complete identity of the inhibitory neurotransmitters involved in smooth muscle relaxation through the NANC pathway remains unclear in the literature (Richardson et al., 2023).

In addition to tonic contractions, the PS exhibits phasic contractions regulated by ICCs, which share similar properties with those in the antrum of the stomach (López-Pingarrón et al., 2023; Won et al., 2005; Lüdtke et al., 1991). Like the antrum and fundus, the PS comprises circular and longitudinal muscle layers that exhibit elastic properties for storing passive stress (Hur et al., 2021). Changes in basal tone can induce passive stress, which, in turn, may influence the active stress generated by the contractile elements in the muscle.

**Figure 4:**
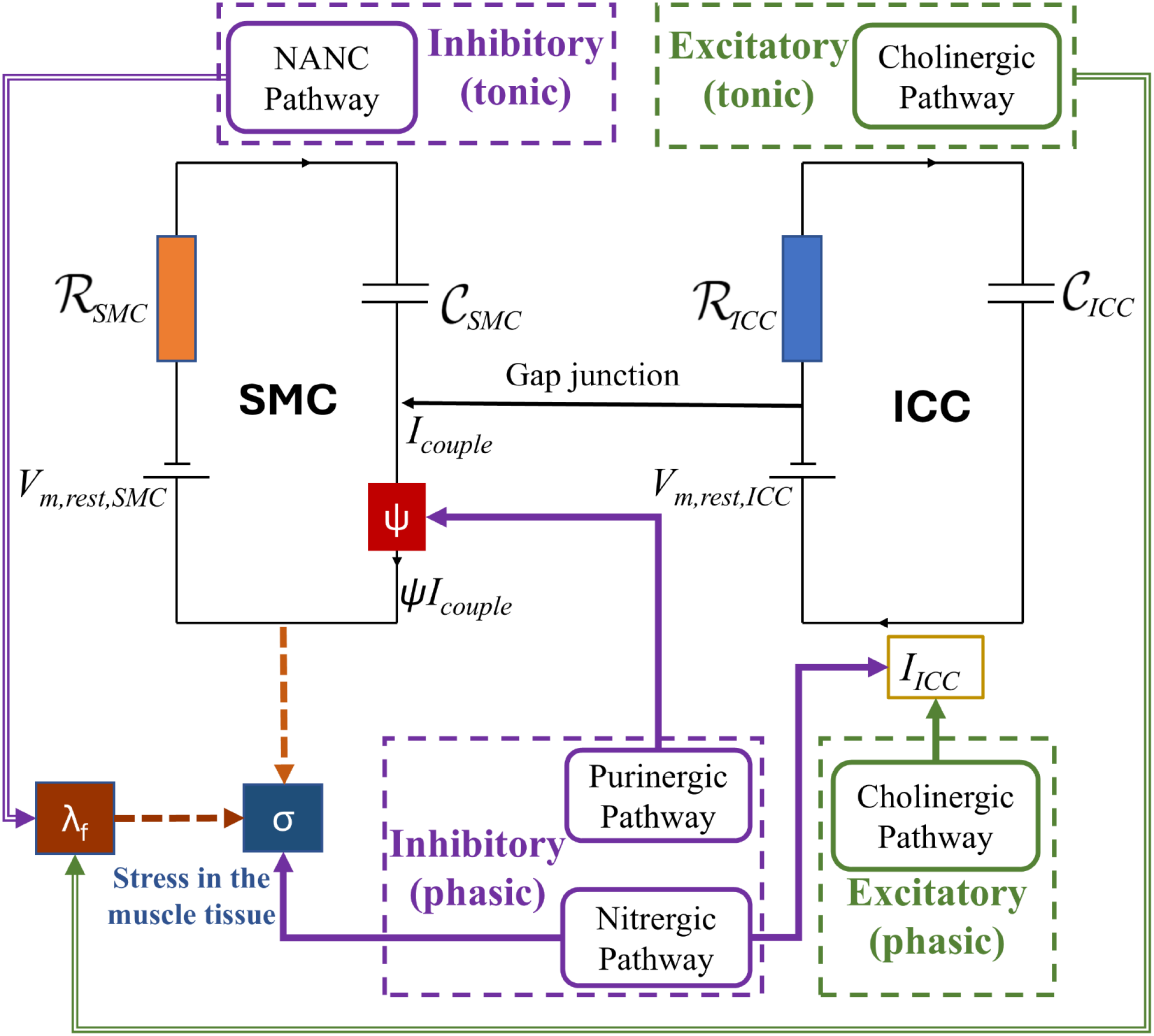
Schematic representation of excitatory and inhibitory stimulation for PS phasic and tonic activity. Smooth muscle cells (SMC) and interstitial cells of Cajal (ICC) are represented as electrically coupled compartments connected by gap-junction current *I_couple_*. The excitatory cholinergic tonic pathway and the inhibitory non-adrenergic non-cholinergic (NANC) tonic pathway influences the contractile-element deformation parameter *λ_f_*. These tonic effects contribute to tissue stress *σ*. In the phasic pathway, excitatory cholinergic input modulates ICC activity through *I_ICC_*, while inhibitory purinergic and nitrergic pathways regulate ICC and SMC responses. Here, ℛ denotes membrane resistance, 𝒞 denotes membrane capacitance, *V_m,rest_* denote the resting membrane potential, respectively, and *ψI_Icouple_* denotes the modulation term associated with the gap-junction coupling current.

For modeling purposes, the signaling framework developed for phasic contractions in the antrum is adapted for the PS. For tonic activity, separate pathways for cholinergic and NANC signaling will be derived. The schematic for the phasic and tonic activity in the PS is shown in **Figure 4**.

Schematic of excitatory and inhibitory regulation of gastric tonic and phasic activity.

#### 2.2.10 Cholinergic pathway: PS

For tonic-controlled activity, due to the lack of data in the literature regarding the effect of the cholinergic pathway on PS contractions, the modeling approach for this pathway is based on equations derived for tonic activity in the fundus. These equations are represented as Equations 2 and 3, where *f_e,tnc,_*_3_ denotes the excitatory firing frequency that regulates tonic activity.

For phasic-controlled activity, the cholinergic pathway neuron is modeled similarly to that of the antrum, as the ICCs in the PS exhibit properties comparable to those in the antrum. Here, the excitatory firing frequency governing phasic activity is represented as *f_e,p,_*_3_.

#### 2.2.11 NANC pathway: PS

For tonic-controlled activity, the identity of the neurotransmitter responsible for inhibitory activity is unknown (Richardson et al., 2023). Therefore, the MMEHC is used directly to model the inhibitory response, where *f_i,tnc,_*_3_ represents the inhibitory firing frequency, and *ϕ_ih,tnc,_*_3_ denotes the inhibitory fraction. The phenomena is modeled using Equation 45, and represented as.

(45) $$\phi_{ih,tnc,3}=\frac{A_{ih,tnc,3}f_{i,tnc,3}^{N_{ih,tnc,3}}}{B_{ih,tnc,3}^{N_{ih,tnc,3}}+f_{i,tnc,3}^{N_{ih,tnc,3}}}$$

Data from (Ishiguchi et al., 2000) was used to estimate the parameters for the equation above.

For phasic activity, the NANC pathway is modeled similarly to that of the antrum, where the inhibitory firing frequency is represented as *f_i_*,*_p_*_,3_.

#### 2.2.12 Equations in the compartmental model for PS

For tonic activity, the intracellular calcium concentration in the SMC is modeled using Eq. 7. The model by (Gajendiran and Buist, 2011) is employed to convert ${\left[Ca\right]}_{SMC}^{2+}$ into active MLCK, denoted as [*MLCK_act_*]*_tnc,_*_3_.

Due to the fact that the neurotransmitter involved in the NANC pathway is unknown, the pathway is modeled such that *ϕ_ih,tnc,_*_3_ increases MLCP concentration and reduces effective MLCK activity, reflecting effects observed in inhibitory pathways (Yang et al., 2005). The MLCK and MLCP concentrations associated with the inhibitory pathway are modeled using Equations 46 and 47 as follows.

(46) $$[MLCP{]}_{tnc,3}=[MLCP{]}_{rest}+\phi_{ih,tnc,3}{\left[MLCP\right]}_{f,tnc,3}$$

(47) $$[MLCK{]}_{tnc,3}=\left(1-\phi_{ih,tnc,3}\right)[MLCK_{act}{]}_{tnc,3}$$

To model the effects of MLCP concentration and effective MLCK activity on tissue deformation due to passive stress (tonic activity), Equations 10–16 are utilized, with detailed explanations provided in **Supplementary Material Section 1.2.1**.

For phasic activity in the PS, the effects of the cholinergic and NANC pathways on the ICC, SMC, and tissue deformation are modeled using equations similar to those developed for the antrum. The details of the model are explained in **Supplementary Material Section 1.2.2**.

To determine the gastric flow rate through the PS, *Q_flow_*, a simplified flow rate equation is represented by Equation 48 as follows.

(48) $$Q_{flow}=Q_{max,flow}{\left(\frac{r_{fin,3}}{r_{max,3}}\right)}^{2}$$

where *Q_max_*,*_flow_* represents the maximum flow rate through the PS, and *r_max_*_,3_ is the maximum radius the PS can achieve. For further details, refer to **Supplementary Material Section 1.2.3**.

### 2.3 Modeling the afferent/sensory neuron connections

The ascending vagal nerves, also known as vagal afferent fibers, constitute approximately 80% of all vagal fibers (Foley and DuBois, 1937; Asala and Bower, 1986). These sensory neurons detect local chemical and mechanical signals from the GI tract and relay this information to the brainstem for processing. There are two primary categories of vagal afferents: chemosensitive and mechanosensitive. (1) Chemosensitive fibers have peripheral endings that respond to chemical stimuli, such as nutrient content, pH levels, hormones, and immune signals. (2) Mechanosensitive afferents include mucosal endings, intramuscular arrays (IMAs), and intraganglionic laminar endings (IGLEs), which respond to mechanical stimuli like mucosal stroking, muscle distension, and contraction (Kang et al., 2004; Li and Page, 2022).

The stomach is rich in mechanosensitive sensory neurons, including mucosal endings, IGLEs, and IMAs, making it highly responsive to stretch and contractions. When the stomach wall distends due to increased gastric volume, mechanosensitive afferent fibers demonstrate higher firing frequencies. This relationship was evidenced in a study by (Williams et al., 2016).

In contrast, chemosensitive afferents are abundant in the proximal small intestine, where they detect nutrient content and signal the brainstem to modulate gastric tone. For instance, chemosensitive signals can increase the basal tone of the PS to slow gastric emptying, allowing sufficient time for nutrient digestion in the small intestine (Richardson et al., 2023; Waise et al., 2018). In our study, we exclude pHsensitive chemosensitive afferents since the compartmental model framework does not currently incorporate microbiome interactions or chemical reactions affecting gut pH.

From a compartmental modeling perspective, two afferent pathways are considered: (a) a mechanosensitive pathway detecting muscle length stretch, influenced by stomach volume changes; and (b) a chemosensitive pathway that detects the caloric content of gastric ingredients as they pass through the PS into the proximal duodenum.

The firing rates of mechanosensitive and chemosensitive receptors are modeled using linear equations. Linear models are appropriate because muscle stretch and nutrient flow rate changes gradually rather than abruptly. Studies by (Zagorodnyuk and Brookes, 2000; Mahdi et al., 2013) have shown that non-linear models are only necessary for abrupt sensory responses. For instance (Zagorodnyuk and Brookes, 2000), demonstrated a linear relationship between firing rate and tissue stretch under gradual changes, and (Mei, 1978), reported a roughly linear relationship between glucose density and vagal afferent firing.

The mechanoreceptor afferent firing rate *f_mech_*, is modeled by Equation 49 as.

(49) $$f_{mech}=X_{mech,a}\lambda_{f,1}+X_{mech,b}$$

where *X_mech,a_* and *X_mech,b_* are the mechanosensitive fitting constants, fitted using data from (Zagorodnyuk and Brookes, 2000).

For chemoreceptors, nutrient content volumetric flow rate *Q_cal_*, is based on (Kong and Singh, 2008), which reports typical flow rates of 2–4 kcal.min^−1^ during gastric emptying. The maximum calorie flow rate per minute is denoted as *γ_max_*.* A* dimensionless constant Ω accounts for flow lost during the periodic opening and closing of the PS. The calorie content per unit volume of gastric liquid is represented as *g_cal_*. Thus, *Q_cal_* is defined by Equation 50 as.

(50) $$Q_{cal}=\frac{\gamma_{max}\Omega }{g_{cal}}$$

The chemoreceptor afferent firing rate *f_chem_* is modeled by Equation 51 as.

(51) $$f_{chem}=X_{chem,a}Q_{cal}+X_{chem,b}$$

where *X_chem,a_* and *X_chem,b_* are the chemosensitive fitting constants translating sensory activity to afferent firing rates.

The constants *X_chem,a_* and *X_chem,b_* were fitted to ensure that the maximum afferent firing rate was 30 Hz, a value reported by (Mei, 1978). The parameter Ω was determined by analyzing the gastric emptying rate from a dataset in the study by (Kwiatek et al., 2009).

Additionally, the model incorporates an afferent firing response triggered by “fight or flight” conditions. This response is modeled as an on-off (binary) variable $O_{sym}$, where $O_{sym}=1$ indicates the presence and $O_{sym}=0$ indicates the absence of a sympathetic response. When activated, the sympathetic response overrides all parasympathetic pathways, inhibiting gastric emptying by reducing peristaltic activity, maintaining PS closure (Moore and Dalley, 2018; Standring, 2016).

### 2.4 Modeling the interneuron connection in the brainstem

The neurochemically and biophysically diverse second-order neurons of the NTS process sensory information transmitted by vagal afferents. Vagal afferent fibers carry mechanical, chemical, and osmotic signals from the viscera to the NTS, where this information integrates with brainstem, limbic, and hypothalamic inputs to ensure optimal control of stomach reflexes, motility, and emptying (Travagli et al., 2006; Andresen and Kunze, 1994; Brookes et al., 2013; Berthoud et al., 2004).

Glutamate is the primary neurotransmitter used by all vagal afferents, irrespective of their modality or function, to relay information to the NTS. Activation of sensory vagal afferent pathways triggers secondorder NTS neurons via glutamate action on N-methyl-D-aspartate (NMDA) and non-NMDA receptors, initiating reflex activities. These second-order neurons utilize various neurotransmitters to regulate the output of DMV neurons, which govern gastric functions and close the vago-vagal reflex loop (Travagli et al., 2006). The topographic organization of visceral sensory afferents within NTS subnuclei introduces spatial heterogeneity in how sensory information is processed and relayed to the brainstem (Travagli et al., 2006).

The NTS provides key synaptic inputs to DMV neurons, which play a central role in controlling vagovagal responses. Among these inputs, gamma-aminobutyric acid (GABA)-ergic projections are central in modulating in modulating DMV neuronal firing rates, thereby influencing vagal efferent output that regulates gastric tone and motility. Blocking GABAergic transmission between the NTS and DMV using the GABA_A_ antagonist bicuculline has been shown to increase the firing rate of most DMV neurons, resulting in enhanced gastric motility and tone (Travagli and Anselmi, 2016).

The vagal efferent or motor inputs to the stomach by DMV neurons are modulated through local cholinergic and NANC pathways. The interneuronal connections between the NTS and DMV, whether direct or indirect, link the afferent sensory neuron firing frequency to the efferent motor neuron firing frequency. This relationship is modeled using a sigmoid curve equation, borrowing from a similar approach used in cardiovascular regulation (Park et al., 2020). A similar modulation is observed in brainstem interactions within the GI system, where motor neuron firing adapts and fine-tunes in response to sensory neuron activity (Browning et al., 2014; Clyburn and Browning, 2021).

In this study, the equation derived by (Park et al., 2020) is utilized to model the relationship between vagal afferent and vagal efferent mechanosensitive responses. This equation models the passive tissue stretch, caused by stomach volume changes, to the efferent firing rate driving peristaltic activity in the antrum. A detailed explanation of this pathway is provided in Section 2.5. The mechanosensitive afferent-efferent relationship is described by Equation 52 as.

(52) $$f_{e,p,2}=\frac{f_{min,mech}+f_{max,mech}e^{\frac{f_{mech}-f_{mid,mech}}{k_{inter,mech}}}}{1+e^{\frac{f_{mech}-f_{mid,mech}}{k_{inter,mech}}}}$$

For the chemosensitive response, which relates nutrient content flow rate to PS opening, a ninth-order polynomial equation is employed. This choice is based on its superior fit for modeling afferent-efferent responses compared to the equation from (Park et al., 2020). A comprehensive description of this pathway is also provided in Section 2.5. The polynomial equation, denoted by Equation 53 is expressed as.

(53) $$f_{i,tnc,3}=\sum_{s=1}^{9}k_{inter,chem,s-1}f_{chem}^{s}$$

For further details on the brainstem interneuron connection equations, their associated parameters, and the rationale for using higher-order polynomial equations, see **Supplementary Materials Section 1.3**.

### 2.5 Gut-brain axis: sympathetic and parasympathetic regulation of gastric function

The gut–brain axis integrates both sympathetic and parasympathetic pathways, as illustrated in **Figure 1A**). Increased sympathetic drive (sensitive to “fight or flight” response) is generally associated with suppression of digestive activity and reduced gastric motility (Udit and Gautron, 2013; Browning and Travagli, 2014; Travagli and Anselmi, 2016). Consistent with this physiological role, the proposed model represents sympathetic influence through inhibition of gastric motility.

In the antrum, phasic activity is inhibited through the NANC pathway, which suppresses peristaltic activity. In the PS, the frequency of the NANC pathway is reduced, while the cholinergic pathway is upregulated, leading to the closure of the PS. This prevents gastric liquid flow from the stomach to the duodenum. Upon activation of the sympathetic response, the efferent neuron frequencies *f_i,p,_*_2_ and *f_e,p,_*_2_ in the antrum are set to 15 Hz and 0 Hz, respectively. Similarly, the frequencies *f_i,tnc,_*_3_ and *f_e,tnc,_*_3_ in the PS are set to 0 Hz and 10 Hz, respectively. These motor neuron efferent frequencies result in inhibitory gastric emptying by closing the PS and reducing peristaltic activity. The IGP is maintained in the fundus compartment via intramural firing of the NANC pathway, which adjusts gastric volume. A schematic representation of the model for the sympathetic pathway is shown in **Figure 1B**).

Since this study focuses on the gut-brain axis during the gastric emptying phase, the parasympathetic pathway is modeled to regulate gastric functions during this phase. The fundus compartment is connected intramurally via the NANC pathway, which is active during gastric emptying to facilitate adaptive relaxation (or gastric accommodation) (Curro et al., 2008).

A piecewise polynomial equation is used to model the intramural connection of the fundus, establishing the relationship between stomach volume *V_tot_* and the firing frequency of the fundic NANC pathway neurons in the ENS, which plays a crucial role in gastric relaxation. Further details on the use of the piecewise polynomial equation and its estimated parameters to model the intramural connection are provided in **Supplementary Material Section 1.3**. The piecewise polynomial equation, represented as Equation 54 is expressed as.

(54) $$f_{i,tnc,1}=\{\begin{array}{ll} \sum_{s=1}^{8}k_{vol,h,s-1}V_{tot}^{s}, & \text{if }V_{tot}\leq V_{tot,o}\\ \sum_{s=1}^{9}k_{vol,l,s-1}V_{tot}^{s}, & \text{otherwise} \end{array}$$

If the stomach is overly full, stronger peristaltic contractions in the antrum are required, which can be achieved by activating the cholinergic pathway. For gastric emptying, the PS must relax to allow gastric liquid to flow from the stomach to the duodenum. This relaxation is mediated by the NANC pathway. Sensory pathways, both mechanoreceptive and chemoreceptive, relay information about stomach tissue stretch to determine fullness and monitor the gastric meal flow rate from the stomach to the duodenum.

Phasic contractions of the PS, which open and close periodically (approximately 3 cpm) (Simmonds et al., 2024; Carlson et al., 1966), are primarily controlled intramurally. However, they may also receive vagal inputs (Akkermans et al., 1989). These phasic contractions, driven by the ICC, close the PS in response to peristaltic waves reaching the terminal antrum (Fernandes et al., 2024). For simplicity, and due to the limited evidence of direct vagal control over phasic PS contractions, the proposed model assumes that ICC-regulated phasic contractions occur at baseline levels, mediated intramurally through the coordinated antral contractions of the stomach. The local conduction of ICC slow waves from the antrum toward the pyloric region has been previously modeled in an gastric electrophysiology studies (Du et al., 2010; O’Grady et al., 2010; Du et al., 2015; Klemm et al., 2020).

The schematic representation of the parasympathetic pathway used in this study is illustrated in **Figure 1C**).

## 3 Results

The compartmental model developed in this study consisted of approximately 70 ordinary differential equations (ODEs) and 81 algebraic equations. Various ODE solvers in MATLAB (version R2024b), such as ‘ode15s’, ‘ode23s’, and ‘ode23t’, successfully solved the model on a standard office laptop. Among them, the ‘ode15s’ solver demonstrated the most efficient computational performance. The computational time was on the order of seconds for simulating approximately 200 seconds of model dynamics. For example, a 200 s simulation required approximately 11 s of CPU time when solved using the ode15s solver on a standard home laptop (7th-generation Intel i7 processor).

### 3.1 Parasympathetic and sympathetic response

**Figure 5:**
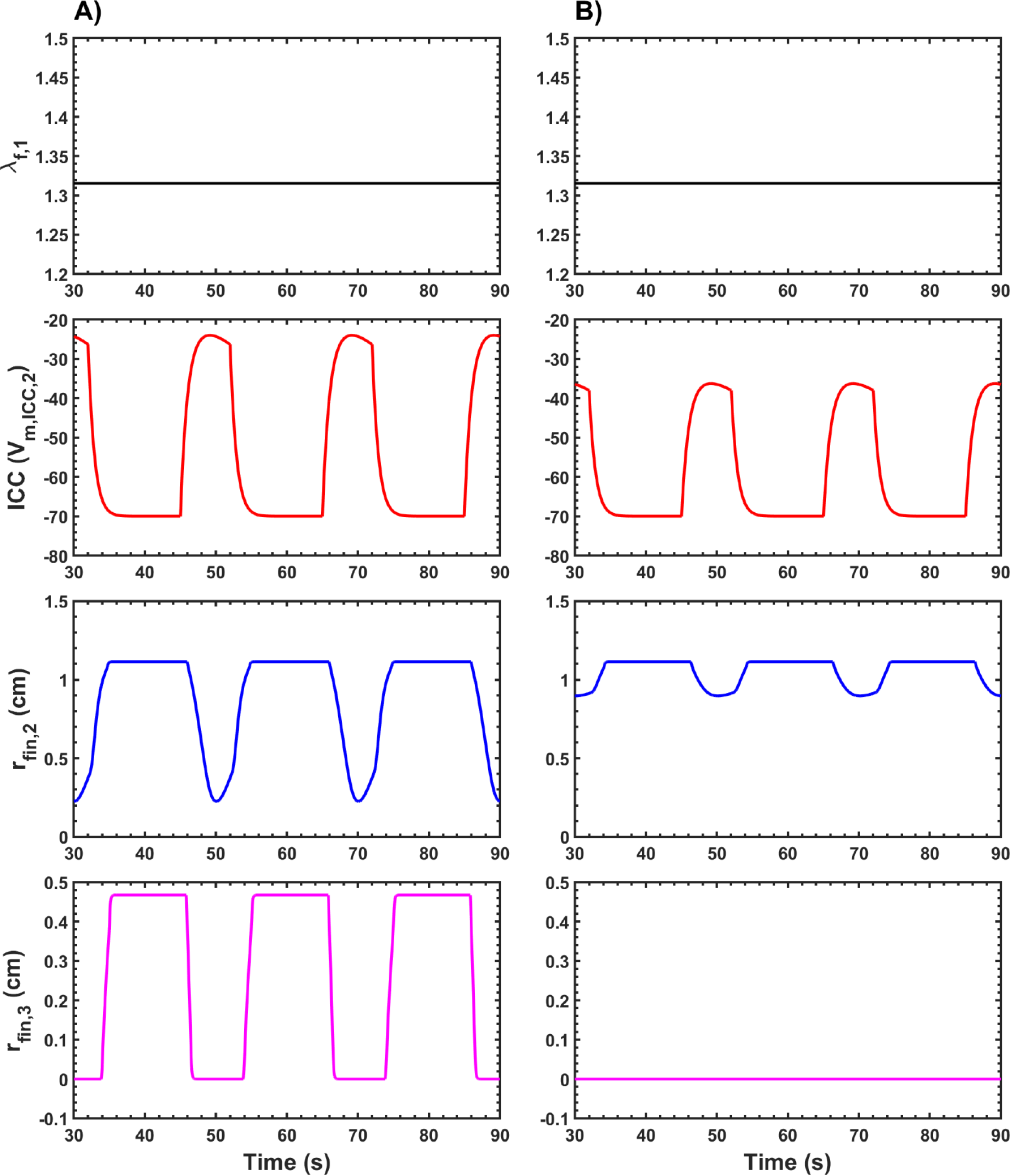
Simulated data showing *λ_f,_*_1_, ICC slow-wave membrane potential *V_m,ICC,_*_2_ (mV), radius of the antrum *r_fin,_*_2_ (cm), and radius of the pyloric sphincter (PS) *r_fin,_*_3_ (cm) over time for **(A)** Parasympathetic and **(B)** Sympathetic responses.

To compute the parasympathetic and sympathetic responses, the total gastric volume (*V_tot)_* was set to 0.6 L. The parasympathetic response was simulated with $O_{sym}=0$, while the sympathetic response used $O_{sym}=1$. The results are shown in **Figure 5**.

The *λ_f,_*_1_ value, which determines fundic relaxation, remained identical between both responses because it depends solely on *V_tot_*, which was constant.

In the antrum, the ICC activity (*V_m,ICC,_*_2)_ was reduced during the sympathetic response compared to the parasympathetic response, leading to weaker peristaltic contractions. During the parasympathetic response, the antral radius (*r_fin,_*_2)_ decreased significantly, showing an occlusion of approximately 79%, while the sympathetic response produced an occlusion of less than 20%.

For the PS, distinct differences were observed between responses. During the parasympathetic response, the PS exhibited periodic opening and closing with a relaxed radius of approximately 0.46 cm. In contrast, during the sympathetic response, the PS remained fully contracted with a radius of 0 cm, preventing gastric outflow.

### 3.2 Fundus and antrum activity during gastric emptying

**Figure 6:**
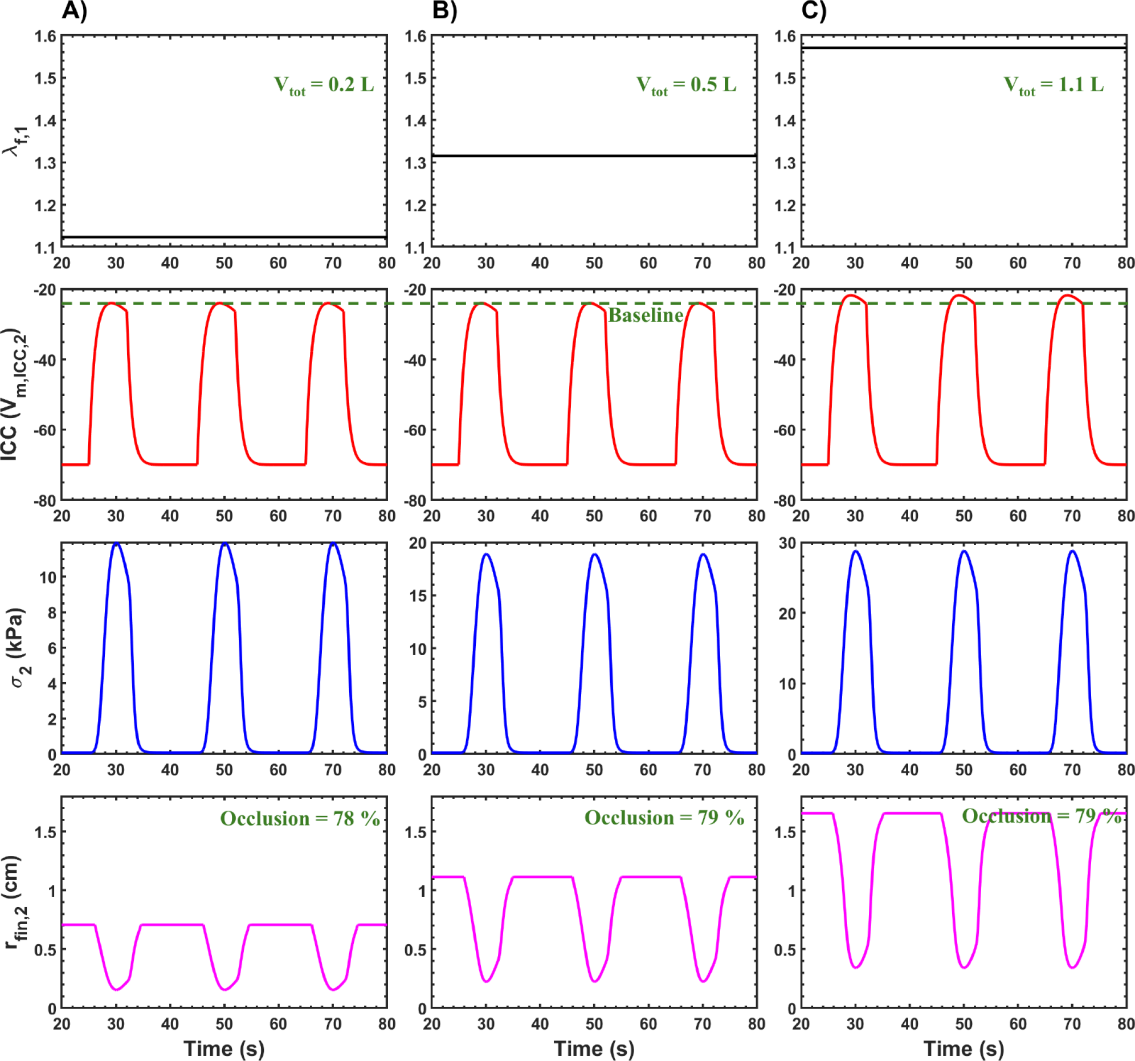
Simulated *λ_f,_*_1_, antral ICC slow-wave potential *V_m,ICC,_*_2_ (mV), tissue stress *σ*_2_ (kPa), and radius *r_fin,_*_2_ (cm) for total gastric volumes *V_tot_* of **(A)** 0.2 L, **(B)** 0.5 L, and **(C)** 1.1 L.

Simulations were performed at three gastric volumes (*V_tot_* = 0.2,0.5,1.1L) to investigate fundic and antral mechanics. The results are shown in **Figure 6**.

As gastric volume increased, the fundic relaxation parameter (*λ_f,_*_1)_ also increased, indicating enhanced relaxation at higher volumes. The amplitude of the antral ICC slow-wave potential (*V_m,ICC,_*_2)_ remained similar at 0.2 L and 0.5 L but increased substantially at 1.1 L.

Antral tissue stress (*σ*_2)_ also increased with *V_tot_*, consistent with stronger muscle activation. Despite the varying gastric volumes, the percentage of antral occlusion remained roughly constant at 78–79%. The pre-contraction antral radius, however, increased with *V_tot_*, approximately 0.7 cm for *V_tot_* of 0.2 L, 1.1 cm (0.5 L), and 1.65 cm (1.1 L). The absolute contraction amplitude also scaled with volume, being approximately 0.55 cm (0.2 L), 0.89 cm (0.5 L), and 1.31 cm (1.1 L).

### 3.3 PS activity for meals of varying caloric content

**Figure 7:**
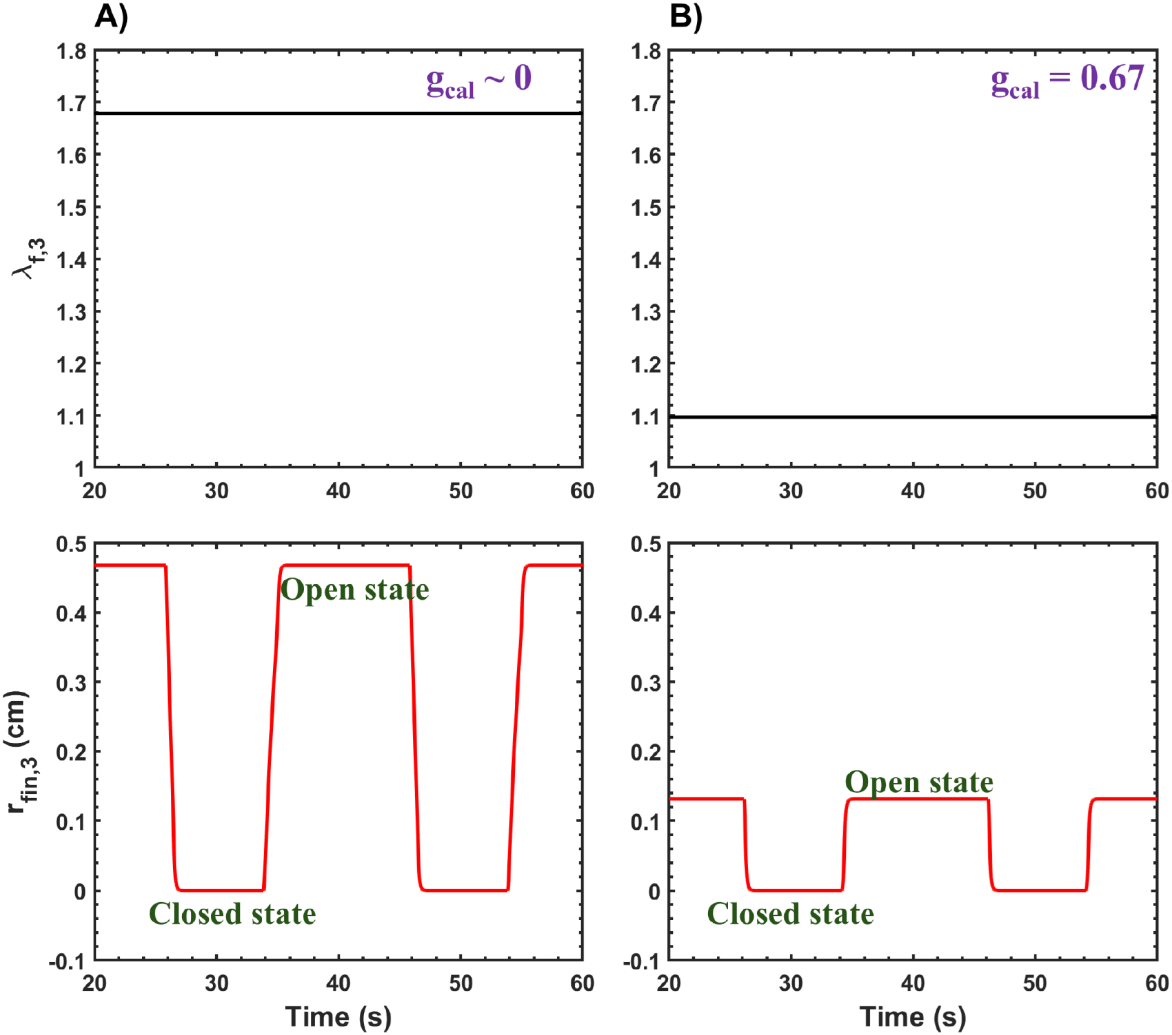
Simulated *λ_f,_*_3_ and pyloric sphincter (PS) radius *r_fin,_*_3_ (cm) over time for gastric liquids with caloric contents of **(A)** 0 kcal.cm^−3^ and **(B)** 0.67 kcal.cm^−3^.

**Figure 7** shows that increasing the *g_cal_* value from 0 to 0.67 decreased *λ_f,_*_3_, indicating greater passive stress and higher basal tone in the PS. Consequently, the PS radius (*r_fin,_*_3)_ during the open state decreased with higher *g_cal_* values, from 0.46 cm at *g_cal_* = 0 kcal.cm^−3^ to 0.13 cm at *g_cal_* = 0.67 kcal.cm^−3^.

### 3.4 Impact of meal caloric content on gastric emptying volume

**Figure 8:**
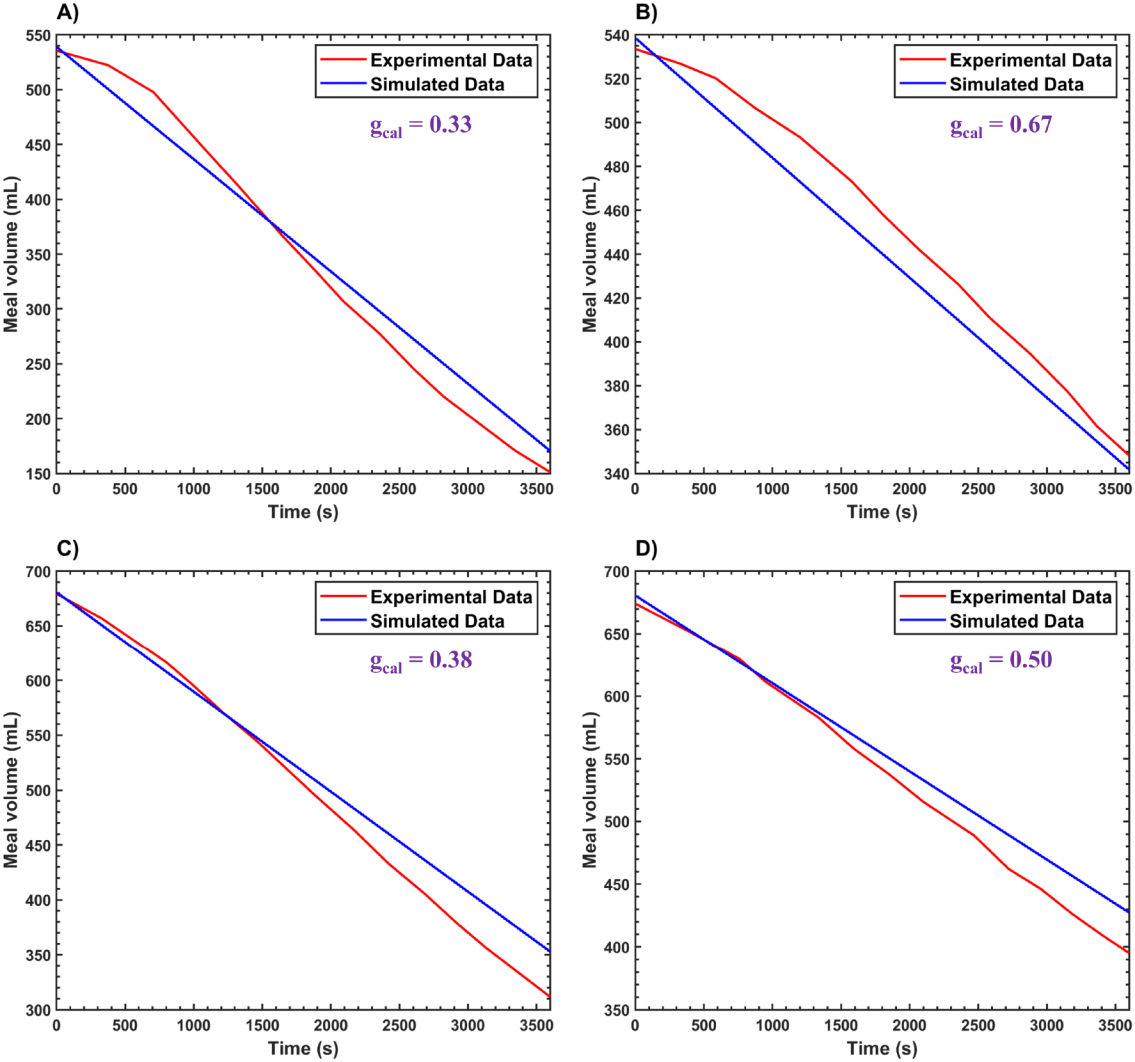
Gastric meal emptying over time for meals of different caloric contents. Experimental data from (Kwiatek et al., 2009) shown alongside simulations for *g_cal_* values of **(A)** 0.33, **(B)** 0.67, **(C)** 0.38, and **(D)** 0.50 kcal.cm^−3^.

Gastric emptying profiles were computed for meals with varying caloric densities (0.33, 0.67, 0.38, and 0.50 kcal.cm^−3^, as shown in **Figure 8**.

The corresponding emptying rates (*Q_flow)_* were approximately 0.1, 0.05, 0.09, and 0.07 mL.s^−1^, respectively. From **Figure 8**, it is observed that higher caloric densities were associated with slower gastric emptying rates.

## 4 Discussion

The model successfully reproduced distinct parasympathetic and sympathetic gastric motor patterns. The parasympathetic mode promoted peristaltic activity and pyloric relaxation, consistent with efficient gastric emptying. Conversely, the sympathetic mode suppressed antral contractions and maintained pyloric closure, reflecting an inhibitory effect on gastric motility. These findings align with physiological evidence showing that sympathetic activation inhibits gastric motility (Rowbotham et al., 2006; Moore and Dalley, 2018; Standring, 2016).

To clarify the physiological interpretation of the model outputs, each primary output variable corresponds to a measurable or clinically relevant physiological quantity. Although some variables cannot be directly measured *in vivo*, we outline below how the corresponding physiological quantities are assessed experimentally. At present, comprehensive datasets that simultaneously measure all modeled variables are limited; therefore, validation is performed using the best available experimental evidence. The compartment radius represents luminal diameter changes and reflects gastric wall deformation measured using MRI or other imaging-based motility assessments (Sclocco et al., 2021, 2022; Lu et al., 2022). Active and passive stresses represent smooth muscle tension and gastric wall mechanics and relate to experimentally reported measures of compliance, tissue stress, contractility (Kwiatek et al., 2009; Klemm et al., 2020, 2022; Panda and Buist, 2018). The modeled ICC amplitude reflects slow-wave electrical activity and is physiologically linked to experimentally observed slow-wave frequency and amplitude recorded using electrophysiology or high-resolution mapping techniques (Du et al., 2010; O’Grady et al., 2010, 2014). The gastric emptying rate directly corresponds to clinically measured emptying curves obtained via MRI or related imaging modalities allowing quantitative comparison (Sclocco et al., 2022; Kwiatek et al., 2009; Sclocco et al., 2021).

In the fundus and antrum, the simulations captured physiologically realistic volume-dependent mechanical behavior. Increasing gastric volume led to elevated fundic relaxation (*λ_f,_*_1)_ and amplified antral contraction amplitudes, consistent with adaptive relaxation and the length–tension relationship observed experimentally (Kindt and Tack, 2006; Mundt and Samsom, 2006; Tack et al., 1998; Murtada et al., 2012). The constant occlusion ratio across volumes supports the idea of a regulated contraction amplitude that scales with luminal load (Perlas et al., 2009; Stemper and Cooke, 1975).

Simulations examining pyloric function across different meal caloric contents showed that higher-calorie liquids increased basal tone (lower *λ_f,_*_3_ leading to and smaller *r_fin,_*_3_ value), limiting gastric outflow. This matches experimental findings showing that calorie-dense meals increase cholecystokinin (CCK) release, enhancing pyloric tone (Richardson et al., 2023; Friedenberg et al., 2008; Schneeman et al., 2003; Gibbons et al., 2016). CCK is a GI hormone released in response to nutrient exposure which plays a key role in modulating vagal afferent signaling and pyloric tone. In the present framework, the multiple neural and hormonal pathways through which CCK influences gastric motility are represented using a phenomenological systems-level formulation, allowing the dominant physiological effects to be captured without resolving each individual pathway (Richardson et al., 2023; Schneeman et al., 2003).

Finally, the model reproduced experimentally observed relationships between meal caloric content and gastric emptying rates. Higher *g_cal_* values yielded slower emptying rates, in agreement with studies by (Kwiatek et al., 2009). This demonstrates the capacity of the model to capture neurogastrointestinal control of gastric motility through integrated electrical, mechanical, and autonomic signaling components.

## 5 Conclusion

The model developed in this study simulates the vago-vagal loop that controls gastric function using a computationally efficient compartmental framework. This framework relies solely on ordinary differential equations (ODEs) and algebraic equations, as proposed in our prior work (Fernandes et al., 2024). The computational efficiency of the model enables it to be executed on a standard office laptop, making it highly accessible. Unlike previous efforts, which have modeled specific components of the gut-brain axis, this study represents the first attempt to comprehensively model the entire ANS regulating gastric function. This study presents a phenomenological yet detailed model of the vago-vagal loop, making it relevant in developing closed-loop therapies for treating GI diseases using concepts from learning and control theory. This is particularly significant given the prevalence of gut-brain axis dysfunction in conditions such as functional dyspepsia (Sclocco et al., 2022).

The main features and contributions of the developed model are summarized as follows.

1. The model uses a compartmental model framework which relies solely on a system of coupled ODEs and algebraic equations, making it lightweight and easy to simulate on standard hardware.
2. Our study presents the first attempt to develop a full systems-level mathematical model that comprehensively captures the vago-vagal reflex loop, including both afferent and efferent pathways, to describe the closed-loop feedback regulation of gastric function during gastric emptying.
3. The model primarily represents the parasympathetic pathway, as it constitutes the dominant neural mechanism governing gastric function during digestion.
4. The proposed framework extends our prior gastric model (Fernandes et al., 2024) by incorporating dynamic changes in gastric volume, modulated by neural firing, rather than assuming a fixed gastric geometry.
5. The framework is designed to be compatible with model-based control architectures, such as closed-loop vagal nerve stimulation. Its potential application in adaptive, patient-specific digital twin–based controllers represents a future direction and will require further research, including model simplification, parameter identifiability analysis, and uncertainty quantification.
6. The approach provides a flexible foundation for multiscale extensions to the model to include sensory feedback, electrogastrography, spatial heterogeneity or coupling to higher autonomic centers in the deeper brain region.

### 5.1 Limitations

Despite the comprehensive structure of the proposed framework, the current model adopts several simplifying assumptions. Spatial dependence and heterogeneity in neural anatomy are neglected. These include the explicit effects of conduction pathways, axonal delays, and distributed neural connectivity which are lost when using the MMEHC equations to model neural activity. Similarly, the fluid mechanics within the stomach have been simplified since the antrum is represented as a single compartment, which limits the spatial resolution of gastric flow and pressure gradients. Furthermore, while the parasympathetic (vagal) pathway is modeled in detail, the sympathetic pathway which plays a key role in inhibiting digestion is only coarsely represented. In the current formulation, autonomic control is approximated using two dominant modes; however, *in vivo*, parasympathetic and sympathetic outflows vary continuously and can be simultaneously active. A more complete model would require inclusion of the sensory feedback mechanisms that initiate sympathetic activity and an explicit representation of the parasympathetic–sympathetic balance. However, such extensions are currently constrained by the limited availability of experimental data describing sympathetic control of gastric function.

### 5.2 Future work

Potential fruitful directions of future study could include conducting targeted experiments to better characterize the sympathetic response in gastric regulation, particularly its inhibitory effects mediated through sensory and central feedback. Additional directions include developing spatially detailed conduction models to capture efferent and afferent pathway dynamics, refining fluid–structure interactions within gastric compartments, and validating model predictions against experimental data. An important next step will be to perform systematic sensitivity and identifiability analyses to assess the relative contribution of model states and parameters to key physiological outputs. Such analyses will enable principled model reduction, helping to determine which components of the current high-dimensional formulation are necessary to preserve essential behaviors, and which may be simplified to yield a more parsimonious and identifiable model suitable for predictive and patient-specific applications. Finally, the framework can be extended for integration into a model-based closed-loop control system for vagal stimulation therapy (Yao and Kothare, 2020; Yao et al., 2023; Yao and Kothare, 2024).

## Data Availability

The original contributions presented in the study are included in the article/**Supplementary Material** (https://github.com/shanferns/Gut-brain-axis-compartment-model.git). Further inquiries can be directed to the corresponding author.

## Supplementary material

The supplementary material containing detailed derivations and explanations of the equations is provided in the Supplementary Information.
