# Supplementary material for: A compartmental model for simulating the gut-brain axis in gastric function regulation
Source: Front Physiol. 2026 Jun 3;17:1727491. doi: 10.3389/fphys.2026.1727491 (PMC13272412; doi:10.3389/fphys.2026.1727491)
Supplement: Supplementary file 1 [file DataSheet1.pdf]

frontiers

Supplementary Material
For the manuscript *"A Compartmental Model for Simulating the Gut-Brain Axis in Gastric Function Regulation"*, by S. Q. Fernandes, M. V. Kothare

1 DETAILED DERIVATIONS AND EXPLANATIONS OF NECESSARY EQUATIONS

1.1 Applying the Michaelis-Menten equation with a Hill coefficient (MMEHC) equation to model neuronal firing effects on smooth muscle responses

To apply the MMEHC equation, neurotransmitter release following neuronal firing is analyzed. Specifically, the firing dynamics of a dorsal motor nucleus of the vagus (DMV) neuron projecting to the stomach is modeled. The action potential of the DMV neuron is simulated using Hodgkin–Huxley-type equations, with most ion channel gating kinetics following the framework outlined in (Briant et al., 2014). Modifications were introduced to simplify the model representation of the DMV neuron.

The calcium concentration at the synaptic cleft, resulting from DMV neuron action potentials, is modeled using equations from (Erler et al., 2004). The corresponding neurotransmitter release, driven by synaptic calcium concentration, is modeled using a mathematical framework from (Briant et al., 2015). The relationship between DMV action potential firing frequency and normalized neurotransmitter release is then plotted, and the MMEHC equation is fitted to these data for analysis.

For modeling neurotransmitter release and its effect on smooth muscle response—such as changes in contractile force—the MMEHC equation can be applied based on (Briant et al., 2015). The plots in this study demonstrate that these dynamics follow non-linear saturation curves, which the MMEHC equation effectively captures. Moreover, the relationship between neurotransmitter release and smooth muscle response resembles a lumped ligand-receptor binding mechanism, a type of interaction that the MMEHC equation is well-suited to model.

1.1.1 Neuron modeling: Action potential in the DMV

The governing equation for the membrane voltage of the DMV neuron, $V_{m,DMV}$, is given by

$$\frac{dV_{m,DMV}}{dt} = \frac{-I_{ion,DMV} + I_{stim,DMV}}{C_{m,DMV}} \tag{S1}$$

Here, $I_{ion,DMV}$ represents the total ionic current in the DMV neuron, computed as the sum of sodium, potassium, calcium, and leak currents, derived from the dynamics of voltage-gated channels. $I_{stim,DMV}$ denotes the external stimulation current applied to the DMV neuron. Most ionic channels are modeled following the approach of (Briant et al., 2014). However, certain parameters, such as the conductance of specific ion channels, were manually adjusted to better fit the experimental action potential data of DMV neurons projecting to the stomach, as reported by (Browning et al., 2005).

$$I_{ion,DMV} = I_{Na,DMV} + I_{KDR,DMV} + I_{Pas,DMV} + I_{KCa,DMV} + I_{CaL} + I_{CaN} \tag{S2}$$

The leak current, $I_{Pas,DMV}$, modeled similarly to (Briant et al., 2014), is given by

$$I_{Pas,DMV} = g_{Pas}(V_{m,DMV} - E_L) \tag{S3}$$

The calcium activated potassium current, $I_{KCa,DMV}$, modeled similarly to (Briant et al., 2014), is denoted by

$$I_{KCa,DMV} = g_{KCa}o_{KCa}(V_{m,DMV} - E_K) \tag{S4}$$

The delayed rectifier current, $I_{KDR,DMV}$, modeled similarly to (Briant et al., 2014), is represented by

$$I_{KDR,DMV} = g_{KDR}n^3_{KDR}l_{KDR}(V_{m,DMV} - E_K) \tag{S5}$$

The sodium current, I_{Na}, is modeled similar to (Briant et al., 2014). However, the channel representation was simplified to a traditional sodium channel with only two gating variables. The activation ($m_{Na,DMV}$) and inactivation ($h_{Na,DMV}$) gating variables follow the same formulation as described by Briant et al., 2014 (Briant et al., 2014). The sodium current equation is given by

$$I_{Na} = g_{Na}m^3_{Na,DMV}h_{Na,DMV}(V_{m,DMV} - E_{Na}) \tag{S6}$$

The N-type calcium current, I_{CaN}, modeled similar to (Briant et al., 2014), is denoted by

$$\begin{aligned} I_{CaN} = -g_{CaN}m^2_{CaN}\left(\frac{0.001}{0.001+[Ca^{2+}]_{i,DMV}}\right) \\ \times 12.5\left(1 - \frac{[Ca^{2+}]_{i,DMV}}{[Ca^{2+}]_{o,DMV}}\right)\exp\left(\frac{V_{m,DMV}}{12.5}\right)\operatorname{erf}\left(\frac{V_{m,DMV}}{12.5}\right) \end{aligned} \tag{S7}$$

The L-type calcium current, I_{CaL}, is modeled similar to (Briant et al., 2014). However, the intracellular calcium concentration in the DMV neuron is scaled by a factor of 5 to adjust the calcium concentration to match its contribution to the total current in the neuron. The equation is given by

$$\begin{aligned} I_{CaL} = -g_{CaL}m^2_{CaL}\left(\frac{0.001}{0.001+[Ca^{2+}]_{i,DMV}}\right) \\ \times 12.5\left(1 - \frac{5[Ca^{2+}]_{i,DMV}}{[Ca^{2+}]_{o,DMV}}\right)\exp\left(\frac{V_{m,DMV}}{12.5}\right)\operatorname{erf}\left(\frac{V_{m,DMV}}{12.5}\right) \end{aligned} \tag{S8}$$

The potassium activated calcium current, $I_{KCa,DMV}$, modeled similar to (Briant et al., 2014), is denoted by

$$I_{KCa,DMV} = g_{KCa,DMV} o_{KCa} \left(V_{m,DMV} - E_K\right) \tag{S9}$$

The intracellular calcium concentration in the DMV neuron, $[Ca^{2+}]_{i,DMV}$, is modeled using a simplified approach based on (Seydewitz et al., 2017). However, instead of using a Gaussian equation, the conversion from calcium currents to intracellular concentration is derived from (Schild et al., 1994). The equation is given by

$$\frac{[Ca^{2+}]_{i,DMV}}{dt} = -\frac{(I_{CaL} + I_{CaN})A_{DMV}}{FZ_{Ca}V_{DMV}} - k_{\infty}([Ca^{2+}]_{i,DMV} - [Ca^{2+}]_{rest,DMV}) \tag{S10}$$

where

$$V_{DMV} = \frac{4}{3}\pi r_{DMV}^3 \tag{S11}$$

$$A_{DMV} = 4\pi r_{DMV}^2 \tag{S12}$$

Here, r_{DMV} represents the cell radius, V_{DMV} represents the cell volume, and A_{DMV} denotes the cell surface area. The valence of calcium ions is given by Z_{Ca}, and F is Faraday's constant. The resting intracellular calcium concentration in the DMV neuron is denoted as $[Ca^{2+}]_{rest,DMV}$.

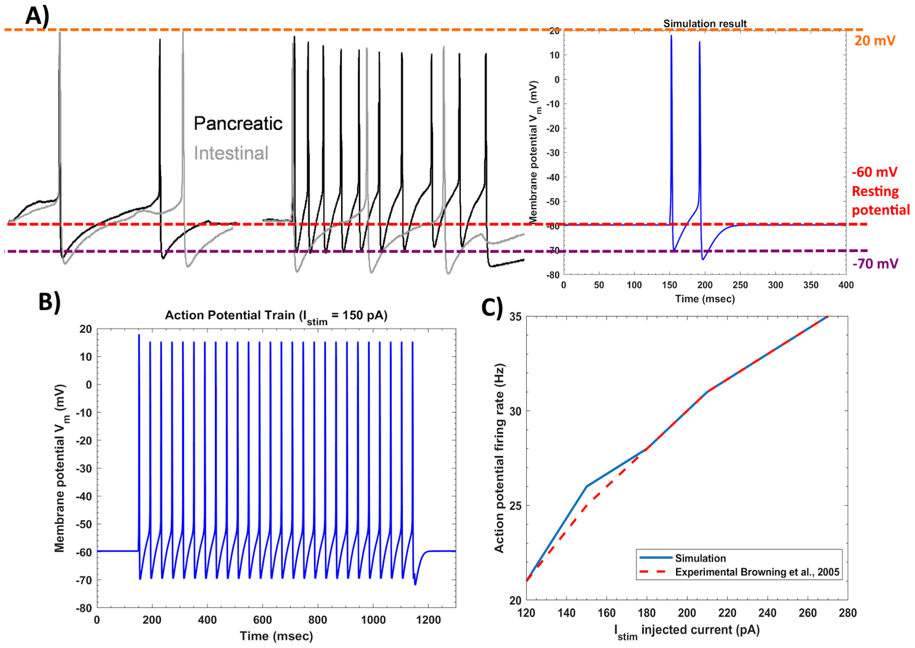

Figure S1: Action potential of DMV neurons projecting to the stomach A) Comparison of shape of action potentials between Experimental (Browning et al., 2005) and simulated by the models proposed in this section B) train of action potentials simulated for external stimulus current $I_{stim} = 150$ pA C) Comparison of experimental (Browning et al., 2005) and simulated action potentials trains for different I_{stim} values

Table S1: Parameters for DMV neuron action potential

Parameter	Value	Unit	Reference
g_{Pas}	0.26	μS.cm^{-2}	Chosen
g_{Na}	40	μS.cm^{-2}	Chosen
g_{CaL}	1	μS.cm^{-2}	Chosen
g_{CaN}	8	μS.cm^{-2}	Chosen
g_{KDR}	2	μS.cm^{-2}	Chosen
g_{KCa}	10	μS.cm^{-2}	Chosen
$[Ca^{2+}]_{rest,DMV}$	50	nM	Migliore et al. (1995)
r_{DMV}	12	μm	Browning et al. (2005)
Z_{ca}	2	mV	Schild et al. (1994)
E_K	-80	mV	Chosen
E_L	-40	mV	Briant et al. (2014)
E_{Na}	20	mV	Chosen
k_∞	2	ms^{-1}	Chosen
$C_{m,DMV}$	1	μF.cm^{-2}	Migliore et al. (1995)

1.1.2 Calcium concentration in the synaptic cleft

The calcium concentration in the synaptic cleft, which is essential for neurotransmitter release from vesicles in response to an action potential in the DMV neuron, is modeled using a mathematical framework developed by (Erler et al., 2004). The parameters governing this model can be found in their study.

The time-dependent single-channel open probability, g_v, is described by

$$\frac{dg_v}{dt} = \frac{\hat{g}_v - g_v}{\tau} \tag{S13}$$

where τ is the time constant.

The calcium concentration in the synaptic cleft of the DMV neuron, denoted as $[Ca^{2+}]_{free}$, evolves according to

$$\frac{d[Ca^{2+}]_{free}}{dt} = \left(\frac{[G]}{Z_{Ca}F}\right)(J_i - J_e + L)\,\frac{1}{1 + T_{en} + T_{ex}} \tag{S14}$$

The steady-state open probability, $\hat{g}_v$, follows a sigmoidal relationship

$$\hat{g}_v = \frac{1}{\exp\left(\frac{V_h - V_{m,DMV}}{\epsilon}\right) + 1} \tag{S15}$$

where ϵ represents the steepness of activation, and V_h is the half-activation voltage.

The inward current, I_{open}, associated with channel opening due to an action potential in the DMV neuron, is defined as

$$I_{\text{open}} = \begin{cases} 0, & V_{m,DMV} > \bar{V}_c \\ \bar{g}_v(\bar{V}_c - V_{m,DMV}), & \text{otherwise} \end{cases}$$

where $\bar{V}_c$ is given by

$$\bar{V}_c = \left(\frac{RT}{zF}\right) \ln\left(\frac{[Ca]_{ext}}{[Ca^{2+}]_{free}}\right) - \Delta V_{eff} \tag{S16}$$

The calcium influx current density, J_i, is defined as

$$J_i = \rho_v g_v I_{\text{open}} \tag{S17}$$

For the complete set of model equations, refer to (Erler et al., 2004). The simulated synaptic cleft calcium concentration for different action potential frequencies in the DMV neuron is presented in Fig. S2.

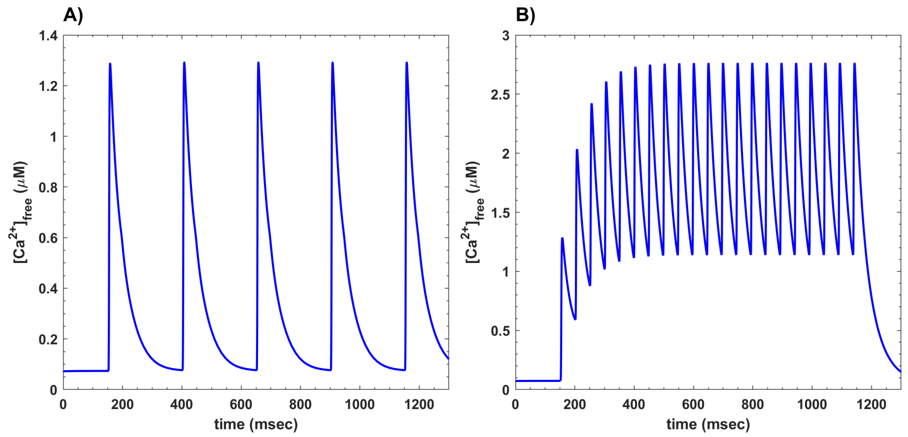

Figure S2: Calcium concentration in the DMV neuron synapse $[Ca^{2+}]_{free}$ for different firing frequency of DMV neuron action potential firing frequency A) 4 Hz B) 21 Hz

1.1.3 Neurotransmitter release

The concentration of neurotransmitter release, $[NA]$, in response to calcium concentration at the synaptic cleft, $[Ca^{2+}]_{free}$, is simulated using the model from (Briant et al., 2015). The parameters for this model are detailed in that study. However, the calcium ion binding rate constant, k_b, was varied with values of $k_b = 10^{14}\,\text{mM}^{-4}.\text{ms}^{-1}$, $k_b = 10^{13}\,\text{mM}^{-4}.\text{ms}^{-1}$, and $k_b = 10^{12}\,\text{mM}^{-4}.\text{ms}^{-1}$. The corresponding simulation results are shown in Fig. S3. This variation was introduced to assess the sensitivity of neurotransmitter release to different rates of calcium-mediated protein binding.

The equations governing (Briant et al., 2015) model are:

$$\frac{d[F_A]}{dt} = k_b(F_{max} - [F_A] - [V_A])[Ca^{2+}]^4_{free} - k_u[F_A] - k_1[F_A]V + k_2[V_A] \tag{S18}$$

$$\frac{d[V_A]}{dt} = k_1[F_A]V - (k_2 + k_3)[V_A] \tag{S19}$$

$$\frac{d[N_A]}{dt} = Nk_3[V_A] - k_h[NA] \tag{S20}$$

From Fig. S3, it can be observed that fitting a MMEHC curve to the simulated data—relating neuronal firing frequency to neurotransmitter release—demonstrates a close alignment with the expected behavior. This suggests that the MMEHC equation is an appropriate model for capturing this relationship.

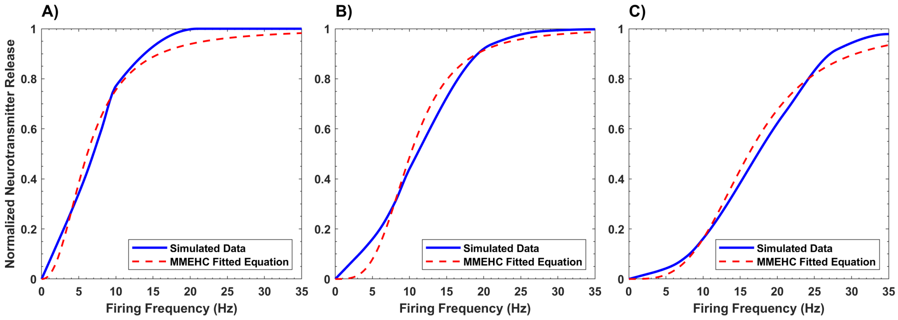

Figure S3: Comparison of simulated data from the model with the MMEHC equation fit for normalized neurotransmitter release concentration as a function of DMV neuron firing frequency. (A) $k_b = 10^{14}\,\text{mM}^{-4}.\text{ms}^{-1}$, (B) $k_b = 10^{13}\,\text{mM}^{-4}.\text{ms}^{-1}$, (C) $k_b = 10^{12}\,\text{mM}^{-4}.\text{ms}^{-1}$.

1.2 Efferent neuron and stomach compartment parameters

1.2.1 Fundus

The MMEHC equation is used to model neurotransmitter release based on the firing frequency of inhibitory and excitatory neurons. Additionally, this equation modulates the relaxation or contraction response induced by neurotransmitter release in the fundus through signaling pathways. The relaxation or contraction response is computed as values ranging from 0 to 1, with the equations derived using the surface area of an open cylinder.

The relaxation response is given by

$$relax = \frac{r_{fin,1} - r_{min,1}}{r_{max,1} - r_{min,1}} \tag{S21}$$

The contraction response is then computed as

$$contract = 1 - relax \tag{S22}$$

Here, $r_{min,1}$ is determined by modeling the stomach as a cylinder and calculating its radius when the gastric volume is at a minimum (0.08 L). Similarly, $r_{max,1}$ is computed by assuming the stomach is cylindrical and calculating the radius at the maximum volume (1.2 L). The value of $r_{fin,1}$ is obtained using Eq. 15.

The parameters for the MMEHC equation were estimated using experimental data from (Kim et al., 2020; da Silva et al., 2018) to fit the response of cholinergic neuron firing frequency to neurotransmitter (Acetylcholine (Ach)) release and the subsequent contraction response. Data from (Hayes et al., 1999; Takahashi and Owyang, 1995; Jenkinson and Reid, 2000; Curro et al., 2008) were used to fit the response of non-adrenergic, non-cholinergic (NANC) neuron firing to neurotransmitter (Nitric oxide (NO) and Vasoactive intestinal peptide (VIP)) release and the corresponding muscle relaxation response.

For parameter fitting, MATLAB 'Curve Fitting' toolbox was utilized, using the 'NonlinearLeastSquares' method with the 'Levenberg-Marquardt' algorithm. Manual adjustments were made to ensure the parameters were physiologically meaningful and produced the appropriate response.

The cholinergic and NANC pathway responses for the fundus are shown in Fig. S4. The response aligns well with experimental data reported in the literature (Hayes et al., 1999; Curro et al., 1994; Takahashi and Owyang, 1995; Kim et al., 2020; Grider et al., 1985). The response for the cholinergic pathway successfully meets the expected contraction response at the desired firing frequency (Fig. S4 A)). For the NANC pathway, the relaxation response is initially dominated by NO when the inhibitory neuron firing frequency is below 0.5 Hz, after which the VIP pathway contributes to the relaxation response in the fundus. This behavior is consistent with previously reported findings (Curro et al., 2008; Currò and Preziosi, 1998; D'Amato et al., 1992; Boeckxstaens et al., 1992).

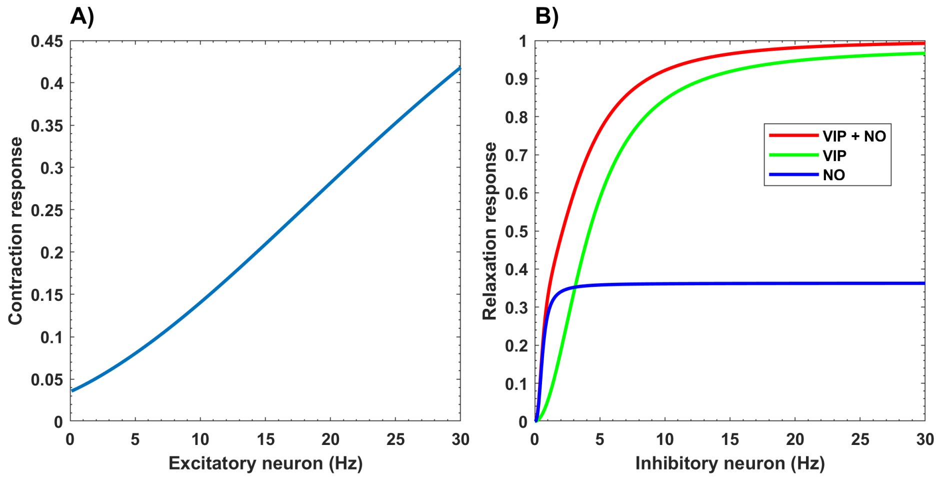

Figure S4: A) Cholinergic neuron firing frequency vs. fundus tissue contraction response when the tissue is fully relaxed ($f_{i,to,1}$ = 15 Hz) B) NANC neuron firing frequency vs. fundus tissue relaxation response. The blue line represents the response for the NO neurotransmitter signaling pathway, the green line represents the response for the VIP neurotransmitter signaling pathway, and the red line represents the combined effect of both pathways

In the fundus muscle tissue, the intracellular calcium concentration at resting state, $[Ca]^{2+}_{rest,1}$, is determined by setting the Smooth Muscle Cells (SMC) membrane voltage to -45 mV (Du et al., 2010) and using the SMC model from (Corrias and Buist, 2007). The $[MLCP]_{max,1}$ value, is assumed to be 7.5 µM, based on the value reported by (Gajendiran and Buist, 2011). This value was chosen as it provides optimal responses for muscle relaxation mediated by NO and VIP neurotransmitter signaling.

The parameter β_1 for the fundus was adjusted to ensure that $r_{fin,1}$ values in Eq. 15 ranged between $r_{min,1}$ and $r_{max,1}$. The dimensionless constant α_1 was computed to maintain $\lambda_{f,1}$ within the range of 1 to 1.7, which is the optimal limit reported by (Panda and Buist, 2021).

1.2.2 Antrum

To model efferent neuron firing in the antrum, data from (Athavale et al., 2024) was primarily used. This study by (Athavale et al., 2024) focused on the inhibitory and excitatory nuron firing responses of Interstitial Cells of Cajal (ICC) and SMC via cholinergic, nitrergic, and purinergic pathways. The data was analyzed based on factors such as ICC and SMC membrane voltage. Fractional values were obtained by normalizing ICC and SMC membrane voltage amplitudes against their baseline values. Similarly, the "slow wave" frequency was normalized against its baseline.

However, additional data were incorporated beyond those reported in (Athavale et al., 2024). Specifically, to model excitatory pathway frequency, data from (Forrest et al., 2006) was used instead of Athavale et al., 2024, as the latter reported minimal changes in "slow wave" frequency at low cholinergic neuron firing rates. To model the impact of the NANC pathway on tissue stress modulation and contraction ratio, data from (Kim et al., 2003) was used. The contraction ratio CR is computed as

$$CR = 1 - \frac{r_{fin,2}}{r_{ini,2}} \tag{S23}$$

where $r_{fin,2}$ and $r_{ini,2}$ are obtained from Eq. 42. Additional data from (Sinn et al., 2010; Forrest et al., 2006; Nakamura and Suzuki, 2004; Kim et al., 2003; Costa et al., 1986) were used to model neuron firing frequency, neurotransmitter release, and their effects on contraction intensity in the antrum.

The MMEHC equation parameters were fitted to model cholinergic and NANC responses in the antrum. The responses for excitatory and inhibitory neuron firing, their effects on contraction ratio, and "slow wave" frequency are shown in Fig. S5. The results align with previous studies, where increasing excitatory neuron firing frequency leads to increased contraction ratio and ICC frequency, consistent with (Athavale et al., 2024). Minimal increases in ICC frequency at low excitatory firing frequencies align with (Forrest et al., 2006). Increasing inhibitory firing frequency reduces the contraction ratio while having little effect on ICC frequency, consistent with (Kim et al., 2003).

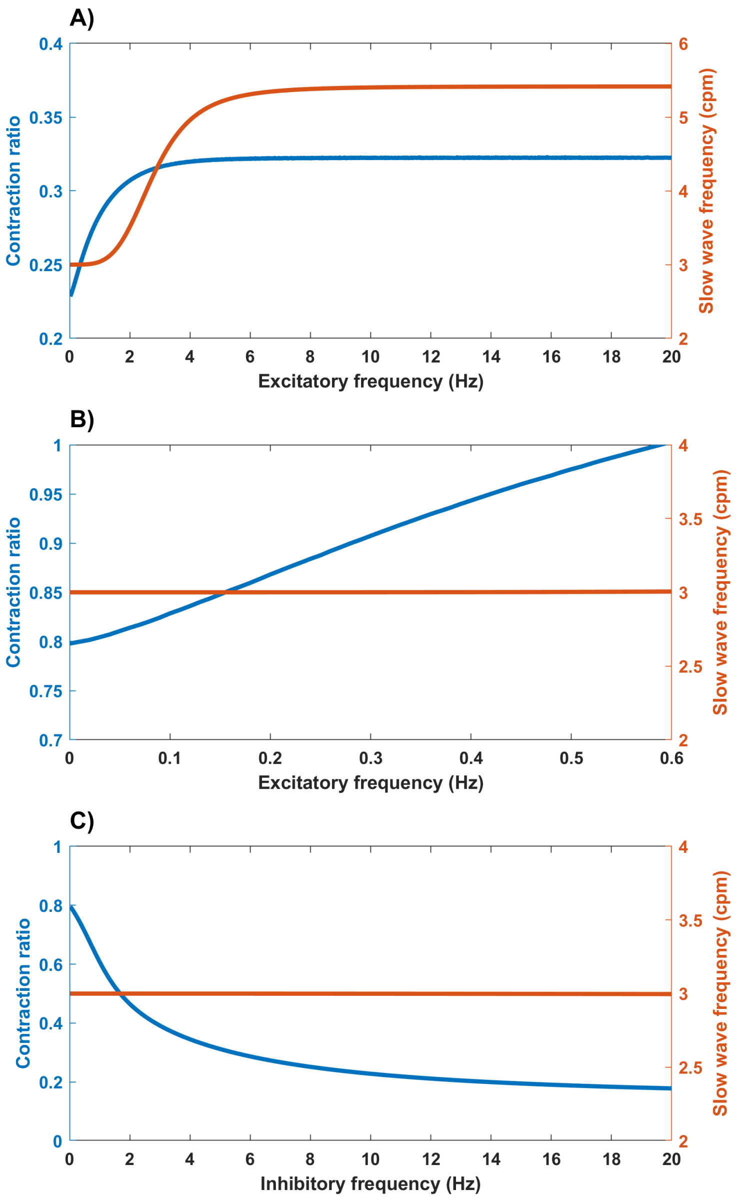

Figure S5: A) Excitatory neuron firing frequency vs. contraction ratio vs. ICC frequency when the tissue is relaxed ($f_{i,p,2}$ = 15 Hz). B) Excitatory neuron firing frequency vs. contraction ratio vs. ICC frequency. C) Inhibitory neuron firing frequency vs. contraction ratio vs. ICC frequency.

To compute tissue stretch, the hyperelastic component of the non-linear viscoelastic model (NLVM) model was modified using a fifth-order polynomial equation, fitted to stress-stretch data from (Panda and Buist, 2021). The choice of a polynomial approach follows the method of (Panda and Buist, 2018), with a fifth-order polynomial selected to capture the highly nonlinear stress-stretch response. The viscoelastic behavior remains modeled using parameters from our previous study (Fernandes et al., 2024).

Table S2. Coefficient values for principal stress E_w

Coefficient	Value
$\mathcal{A}_4$	305.9792
$\mathcal{A}_3$	−2321.3779
$\mathcal{A}_2$	7068.6556
$\mathcal{A}_1$	−10773.4619
$\mathcal{A}_0$	8232.1226

1.2.3 Pyloric sphincter (PS)

To model the tonic efferent response of the PS, a similar approach to that described in Section 1.2.1 was used. Due to the lack of data on the cholinergic response of the PS, data from cholinergic neurons in the fundus were used instead. This substitution was based on experimental findings indicating that the PS exhibits a contraction response to a given Ach concentration similar to that observed in the fundus (Zhao et al., 2016; Vogalis and Sanders, 1990; Cellek and Moncada, 1997).

To model the inhibitory response of the NANC pathway, data from (Ishiguchi et al., 2000) were used. However, the specific inhibitory neurotransmitter for the PS remains unclear (Richardson et al., 2023). As a result, NANC neuron stimulation was modeled as directly influencing inhibitory signaling without explicitly simulating neurotransmitter release.

Using these experimental data, parameters for the MMEHC equation were determined for both the cholinergic and NANC neuron pathways. The corresponding model response is presented in Fig. S6. The contraction and relaxation responses align well with experimental findings (Zhao et al., 2016; Ishiguchi et al., 2000).

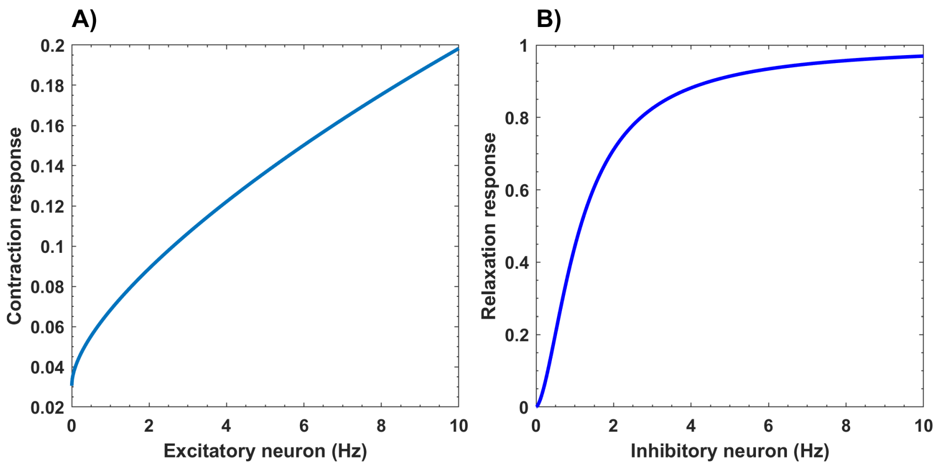

Figure S6: A) Cholinergic neuron firing frequency vs. PS tissue contraction response when the tissue is fully relaxed ($f_{i,to,3}$ = 10 Hz). B) NANC neuron firing frequency vs. PS tissue relaxation response.

To compute the resting intracellular calcium concentration in the PS ($[Ca]^{2+}_{rest,3}$), a method similar to that described in Section 1.2.1 was employed. However, the resting SMC membrane voltage in the PS is reported to be -57 mV (Ward et al., 1998; Hall, 2016), and the corresponding resting intracellular calcium concentration was calculated accordingly.

The parameter β_3 for the PS was adjusted to ensure that $r_{fin,3}$ values in Eq. 15 remained within the range of $r_{min,3} = 0.075$ cm to $r_{max,3} = 0.48$ cm (Hall, 2016; Fernandes et al., 2024; Ishida et al., 2019). Additionally, the value of α_3 was computed to maintain $\lambda_{f,3}$ within the range of 1 to 1.7, as discussed in Section 1.2.1.

To compute the gastric flow rate through the PS, Q_{flow}, a simplified equation is used. Here, $Q_{flow,max}$ represents the maximum flow rate, which occurs when the PS is fully open. The value of $Q_{flow,max}$ is derived from literature data for gastric emptying of a zero-calorie liquid with low viscosity, such as water (Rehrer et al., 1989; Noakes et al., 1991; Fernandes et al., 2024). For such a liquid, gastric emptying occurs at the fastest rate. This flow rate is then adjusted by a factor that accounts for the resistance to gastric flow, which depends on the sphincter radius and the degree of PS occlusion. To keep the model simple, a quadratic relationship is applied, consistent with literature on liquid flow through valves at different opening percentages (Fernandes et al., 2024; Hollingshead et al., 2011; Arun et al., 2015; Alkhulaifi et al., 2023).

1.3 Brainstem and intramural connections

To model the correlation between afferent and efferent neuron signaling (Park et al., 2020) established a relationship between their firing frequencies using a sigmoid function.

For the mechanoreceptor that influences antral contractions based on gastric volume, a similar approach was used as in (Park et al., 2020) to correlate afferent and efferent neuron firing. In this model, the mechanosensitive afferent neuron firing frequency, f_{mech}, was linked to the excitatory cholinergic efferent neuron firing frequency in the antrum, $f_{e,p,2}$. This correlation is justified because, at higher gastric volumes, stronger antral contractions are required to facilitate gastric emptying (Stemper and Cooke, 1975). The antral contractions (terminal antrum) were maintained at approximately 78–80 % (Fernandes et al., 2024; Ishida et al., 2019). Desired values for afferent-efferent neuron firing correlations were obtained to achieve these contraction levels and are plotted in Fig. S7 A). The sigmoid function from (Park et al., 2020) (Eq. 49) was then fitted to the data, as shown in Fig. S7 A).

For the chemoreceptor that influences PS occlusion based on gastric meal caloric content, a similar methodology was used. The intuition behind relating afferent and efferent firing frequencies, as suggested by (Park et al., 2020), was applied. Based on the desired gastric emptying flow rate, the afferent neuron firing frequency was used to regulate the efferent neuron firing rate, which, in turn, controlled the PS radius. The desired correlation data were obtained and plotted in Fig. S7 B). In this model, the chemosensitive afferent neuron firing rate, f_{chem}, was linked to the inhibitory efferent neuron firing rate, $f_{i,to,3}$, since the PS must relax in response to gastric nutrient content to regulate the emptying rate. Since the NANC pathway is responsible for PS relaxation, the pathway was incorporated accordingly. However, as seen in Fig. S7 B), the correlation plot exhibited strong nonlinearity, making the sigmoid function from (Park et al., 2020) unsuitable. Instead, a polynomial function (Eq. 50) was used to capture the afferent-efferent neuron firing relationship while following the intuitive approach from (Park et al., 2020).

For the intramural connection that regulates fundus distension to maintain intragastric pressure (IGP) based on gastric meal volume (as discussed in Section 3.4), gastric volume was estimated using the volume

equation for a cylinder with a fixed height (Fernandes et al., 2024; Gray, 1878, 1924), while varying the radius $r_{fin,1}$. The value of $r_{fin,1}$ was obtained from Eq. 15 by varying the NANC pathway firing frequency $f_{i,to,1}$, which is responsible for fundus relaxation. The resulting data, representing the desired correlation, was plotted in Fig. S7 C). To model this relationship, a piecewise polynomial function (Eq. 51) was fitted to determine the extent of fundus distension required for a desired gastric volume. The fitted piecewise polynomial function and the desired correlation are illustrated in Fig. S7 C).

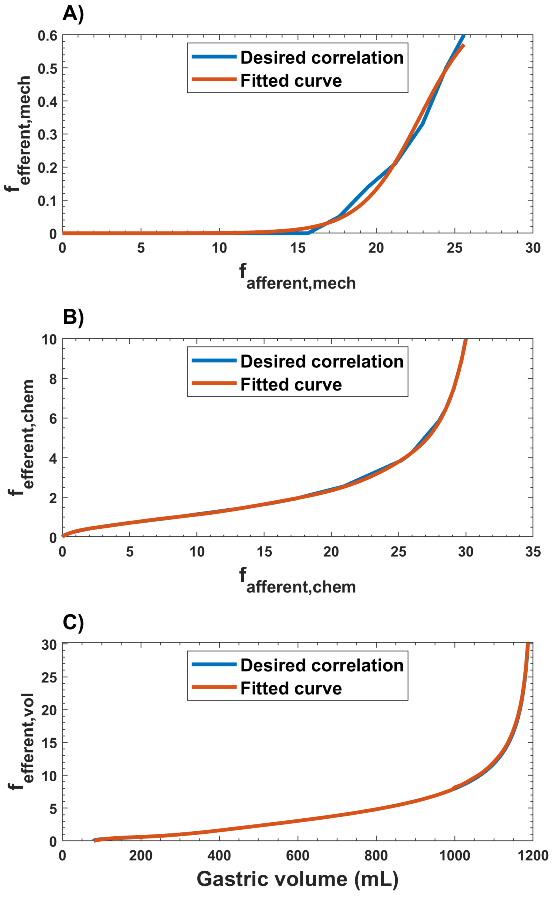

Figure S7: Desired correlation vs fitted curves for A) $f_{afferent,mech}$ (Hz) vs $f_{efferent,mech}$ (Hz) B) $f_{afferent,chem}$ (Hz) vs $f_{efferent,chem}$ (Hz) C) Gastric volume (mL) vs $f_{efferent,vol}$ (Hz)

To justify the use of higher-order polynomial functions beyond visual fit alone, we compared the polynomial representations against sigmoidal functions of the form used by (Park et al., 2020) using both the Akaike information criterion (AIC) and Bayesian information criterion (BIC). For the chemosensitive afferent-efferent relationship shown in Fig. S8 A), the 9th-order polynomial yielded substantially lower values (AIC = -255.66, BIC = -240.40) than the sigmoidal alternative (AIC = -40.88, BIC = -34.77). Likewise, for the volume-dependent response shown in Fig.S8 B), the piecewise polynomial model was also preferred (AIC = -3906.27, BIC = -3813.43) over the sigmoidal alternative based on both AIC and BIC (AIC = 1260.02, BIC = 1279.57). These results indicate that the polynomial formulations provide a better balance between fit quality and model complexity for the present datasets, and therefore support their use in the current study.

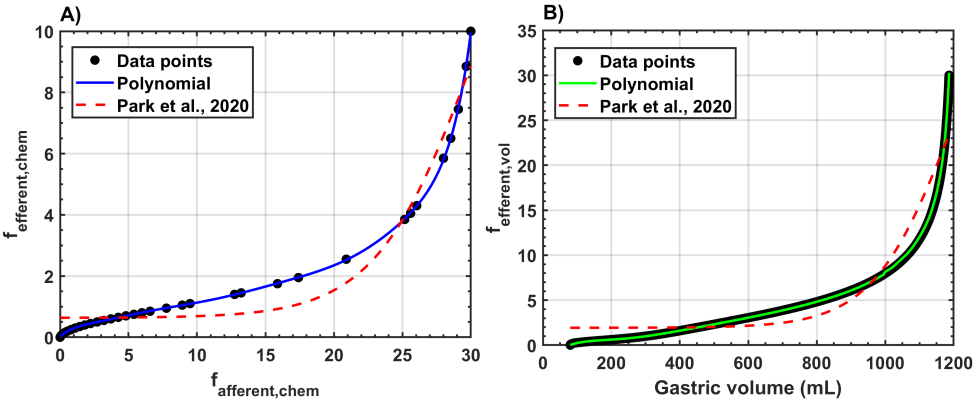

Figure S8: Comparison of polynomial and sigmoidal fitted functions against data points used in the afferent-efferent mapping. A) Chemosensitive afferent input, where the 9th-order polynomial fit is compared with the sigmoidal function adapted from (Park et al., 2020). Black circles denote desired correlation data points. B) Volume-dependent response where the piecewise polynomial fit is compared with the corresponding sigmoidal function. In both cases, the polynomial-based representation more closely follows the desired correlation data points and yielded lower Akaike information criterion (AIC) and Bayesian information criterion (BIC) values than the sigmoidal alternative, indicating a better trade-off between goodness of fit and model complexity for the available data.

Table S3. Parameter values for the mechanoreceptor brainstem interconnection

Parameter	Value
$f_{min,mech}$	0
$f_{max,mech}$	0.7
$f_{mid,mech}$	22.77
$k_{inter,mech}$	1.903

Table S4. Values of $k_{inter,chem,s-1}$

s	$k_{inter,chem,s-1}$
10	2.6286×10^{-10}
9	-3.2650×10^{-8}
8	1.7001×10^{-6}
7	-4.8147×10^{-5}
6	8.0636×10^{-4}
5	-8.1509×10^{-3}
4	4.9024×10^{-2}
3	-1.6874×10^{-1}
2	3.9815×10^{-1}
1	2.1430×10^{-2}

Table S5. Values of $k_{vol,h,s-1}$ and $k_{vol,l,s-1}$

s	$k_{vol,h,s-1}$	$k_{vol,l,s-1}$
10	1.9882×10^{-17}	-
9	-1.9424×10^{-13}	-5.2753×10^{-22}
8	8.4307×10^{-10}	2.7237×10^{-18}
7	-2.1337×10^{-6}	-5.8880×10^{-15}
6	3.4702×10^{-3}	6.9430×10^{-12}
5	-3.7612	-4.8382×10^{-9}
4	2.7166×10^{3}	2.0069×10^{-6}
3	-1.2609×10^{6}	-4.6750×10^{-4}
2	3.4126×10^{8}	5.8051×10^{-2}
1	-4.1033×10^{10}	-2.4861

REFERENCES

Alkhulaifi, K., Alharbi, A., Alardhi, M., Alrajhi, J., and Almutairi, H. H. (2023). Comparative analysis of the performance characteristics of butterfly and pinch valves. *Processes* 11, 1897

Arun, R., Yogesh Kumar, K., and Seshadri, V. (2015). Prediction of discharge coefficient of venturimeter at low reynolds numbers by analytical and cfd method. *International Journal of Engineering and Technical Research (IJETR) ISSN* , 2321–0869

Athavale, O. N., Avci, R., Clark, A. R., Di Natale, M. R., Wang, X., Furness, J. B., et al. (2024). Neural regulation of slow waves and phasic contractions in the distal stomach: a mathematical model. *Journal of Neural Engineering* 20, 066040

Boeckxstaens, G., Pelckmans, P., De Man, J., Bult, H., Herman, A., and Van Maercke, Y. (1992). Evidence for a differential release of nitric oxide and vasoactive intestinal polypeptide by nonadrenergic noncholinergic nerves in the rat gastric fundus. *Archives internationales de pharmacodynamie et de therapie* 318, 107–115

Briant, L. J., Paton, J. F., Pickering, A. E., and Champneys, A. R. (2015). Modelling the vascular response to sympathetic postganglionic nerve activity. *Journal of theoretical biology* 371, 102–116

Briant, L. J., Stalbovskiy, A. O., Nolan, M. F., Champneys, A. R., and Pickering, A. E. (2014). Increased intrinsic excitability of muscle vasoconstrictor preganglionic neurons may contribute to the elevated sympathetic activity in hypertensive rats. *Journal of Neurophysiology* 112, 2756–2778

Browning, K. N., Coleman, F. H., and Travagli, R. A. (2005). Characterization of pancreas-projecting rat dorsal motor nucleus of vagus neurons. *American Journal of Physiology-Gastrointestinal and Liver Physiology* 288, G950–G955

Cellek, S. and Moncada, S. (1997). Nitrergic modulation of cholinergic responses in the opossum lower oesophageal sphincter. *British journal of pharmacology* 122, 1043

Corrias, A. and Buist, M. L. (2007). A quantitative model of gastric smooth muscle cellular activation. *Annals of biomedical engineering* 35, 1595–1607

Costa, M., Furness, J., and Humphreys, C. (1986). Apamin distinguishes two types of relaxation mediated by enteric nerves in the guinea-pig gastrointestinal tract. *Naunyn-Schmiedeberg's archives of pharmacology* 332, 79–88

Curro, D., Ipavec, V., and Preziosi, P. (2008). Neurotransmitters of the non-adrenergic non-cholinergic relaxation of proximal stomach. *European Review for Medical & Pharmacological Sciences* 12

Currò, D. and Preziosi, P. (1998). Non-adrenergic non-cholinergic relaxation of the rat stomach. *General Pharmacology: The Vascular System* 31, 697–703

Curro, D., Preziosi, P., Ragazzoni, E., and Ciabattoni, G. (1994). Peptide histidine isoleucine-like immunoreactivity release from the rat gastric fundus. *British journal of pharmacology* 113, 541

da Silva, L. M., Burci, L. d. M., Crestani, S., de Souza, P., da Silva, R. d. C. M. V. d. A. F., Dartora, N., et al. (2018). Acid-gastric antisecretory effect of the ethanolic extract from arctium lappa l. root: role of h+, k+-atpase, ca 2+ influx and the cholinergic pathway. *Inflammopharmacology* 26, 521–530

D'Amato, M., Curro, D., and Montuschi, P. (1992). Evidence for dual components in the non-adrenergic non-cholinergic relaxation in the rat gastric fundus: role of endogenous nitric oxide and vasoactive intestinal polypeptide. *Journal of the autonomic nervous system* 37, 175–186

Du, P., O'Grady, G., Davidson, J. B., Cheng, L. K., and Pullan, A. J. (2010). Multiscale modeling of gastrointestinal electrophysiology and experimental validation. *Critical Reviews™ in Biomedical Engineering* 38

Erler, F., Meyer-Hermann, M., and Soff, G. (2004). A quantitative model for presynaptic free ca2+ dynamics during different stimulation protocols. *Neurocomputing* 61, 169–191

Fernandes, S. Q., Kothare, M. V., and Mahmoudi, B. (2024). A novel compartmental approach for modeling stomach motility and gastric emptying. *Computers in Biology and Medicine* 181, 109035

Forrest, A. S., Ördög, T., and Sanders, K. M. (2006). Neural regulation of slow-wave frequency in the murine gastric antrum. *American Journal of Physiology-Gastrointestinal and Liver Physiology* 290, G486–G495

Gajendiran, V. and Buist, M. L. (2011). A quantitative description of active force generation in gastrointestinal smooth muscle. *International Journal for Numerical Methods in Biomedical Engineering* 27, 450–460

Gray, H. (1878). *Anatomy of the human body*, vol. 8 (Lea & Febiger)

Gray, H. (1924). *Anatomy of the human body* (Lea & Febiger)

Grider, J., Cable, M., Said, S., and Makhlouf, G. (1985). Vasoactive intestinal peptide as a neural mediator of gastric relaxation. *American Journal of Physiology-Gastrointestinal and Liver Physiology* 248, G73–G78

Hall, J. E. (2016). *Guyton and Hall Textbook of Medical Physiology, Jordanian Edition E-Book* (Elsevier Health Sciences)

Hayes, E., Adaikan, P., Ratnam, S., and Ng, S. (1999). 5-ht4 receptors in isolated human corpus cavernosum? *International journal of impotence research* 11, 219–225

Hollingshead, C. L., Johnson, M. C., Barfuss, S. L., and Spall, R. E. (2011). Discharge coefficient performance of venturi, standard concentric orifice plate, v-cone and wedge flow meters at low reynolds numbers. *Journal of Petroleum Science and Engineering* 78, 559–566

Ishida, S., Miyagawa, T., O'Grady, G., Cheng, L. K., and Imai, Y. (2019). Quantification of gastric emptying caused by impaired coordination of pyloric closure with antral contraction: A simulation study. *Journal of the Royal Society Interface* 16, 20190266

Ishiguchi, T., Takahashi, T., Itoh, H., and Owyang, C. (2000). Nitrergic and purinergic regulation of the rat pylorus. *American Journal of Physiology-Gastrointestinal and Liver Physiology* 279, G740–G747

Jenkinson, K. M. and Reid, J. J. (2000). Evidence that adenosine 5'-triphosphate is the third inhibitory non-adrenergic non-cholinergic neurotransmitter in the rat gastric fundus. *British journal of pharmacology* 130, 1627

Kim, D. M., Khing, T. M., Thein, W., Choi, W. S., Shin, C. Y., and Sohn, U. D. (2020). Signaling pathways underlying changes in the contractility of the stomach fundus smooth muscle in diabetic rats. *Archives of pharmacal research* 43, 666–675

Kim, T., La, J., Lee, J., and Yang, I. (2003). Effects of nitric oxide on slow waves and spontaneous contraction of guinea pig gastric antral circular muscle. *Journal of pharmacological sciences* 92, 337–347

Migliore, M., Cook, E., Jaffe, D., Turner, D., and Johnston, D. (1995). Computer simulations of morphologically reconstructed ca3 hippocampal neurons. *Journal of neurophysiology* 73, 1157–1168

Nakamura, E. and Suzuki, H. (2004). Spontaneous activity and its cholinergic modulation in circular smooth muscle isolated from guinea-pig stomach antrum. *Pflügers Archiv* 449, 205–212

Noakes, T. D., Rehrer, N. J., and Maughan, R. J. (1991). The importance of volume in regulating gastric emptying. *Medicine and Science in Sports and Exercise* 23, 307–313

Panda, S. K. and Buist, M. L. (2018). A finite nonlinear hyper-viscoelastic model for soft biological tissues. *Journal of biomechanics* 69, 121–128

Panda, S. K. and Buist, M. L. (2021). An active finite viscoelastic model for gastric smooth muscle contraction. *BioRxiv* , 2021–01

Park, J. H., Gorky, J., Ogunnaike, B., Vadigepalli, R., and Schwaber, J. S. (2020). Investigating the effects of brainstem neuronal adaptation on cardiovascular homeostasis. *Frontiers in neuroscience* 14, 470

Rehrer, N., Beckers, E., Brouns, F., Ten Hoor, F., and Saris, W. (1989). Exercise and training effects on gastric emptying of carbohydrate beverages. *Med Sci Sports Exerc* 21, 540–9

Richardson, J., Dezfuli, G., Mangel, A. W., Gillis, R. A., Vicini, S., and Sahibzada, N. (2023). Cns sites controlling the gastric pyloric sphincter: Neuroanatomical and functional study in the rat. *Journal of Comparative Neurology* 531, 1562–1581

Schild, J., Clark, J., Hay, M., Mendelowitz, D., Andresen, M., and Kunze, D. (1994). A-and c-type rat nodose sensory neurons: model interpretations of dynamic discharge characteristics. *Journal of neurophysiology* 71, 2338–2358

Seydewitz, R., Menzel, R., Siebert, T., and Böl, M. (2017). Three-dimensional mechano-electrochemical model for smooth muscle contraction of the urinary bladder. *Journal of the Mechanical Behavior of Biomedical Materials* 75, 128–146

Sinn, D. H., Min, B.-H., Ko, E.-j., Lee, J. Y., Kim, J. J., Rhee, J. C., et al. (2010). Regional differences of the effects of acetylcholine in the human gastric circular muscle. *American Journal of Physiology-Gastrointestinal and Liver Physiology* 299, G1198–G1203

Stemper, T. J. and Cooke, A. R. (1975). Gastric emptying and its relationship to antral contractile activity. *Gastroenterology* 69, 649–653

Takahashi, T. and Owyang, C. (1995). Vagal control of nitric oxide and vasoactive intestinal polypeptide release in the regulation of gastric relaxation in rat. *The Journal of physiology* 484, 481–492

Vogalis, F. and Sanders, K. M. (1990). Cholinergic stimulation activates a non-selective cation current in canine pyloric circular muscle cells. *The Journal of Physiology* 429, 223–236

Ward, S. M., Morris, G., Reese, L., Wang, X.-Y., and Sanders, K. M. (1998). Interstitial cells of cajal mediate enteric inhibitory neurotransmission in the lower esophageal and pyloric sphincters. *Gastroenterology* 115, 314–329

Zhao, P., Sun, H.-X., Chu, M., and Hou, Y.-P. (2016). Inhibitory effects of botulinum toxin type a on pyloric cholinergic muscle contractility of rat. *Chin J Physiol* 59, 218–224
